# Molecular evolution of globin genes in Gymnotiform electric fishes: relation to hypoxia tolerance

**DOI:** 10.1186/s12862-017-0893-3

**Published:** 2017-02-13

**Authors:** Ran Tian, Mauricio Losilla, Ying Lu, Guang Yang, Harold Zakon

**Affiliations:** 1 Jiangsu Key Laboratory for Biodiversity and Biotechnology, College of Life Sciences, Nanjing Normal University, Nanjing, 210046 China; 2 Department of Integrative Biology, The University of Texas, Austin, TX 78759 USA; 3 Department of Neuroscience, The University of Texas, Austin, TX 78759 USA; 4 Department of Integrative Biology, Michigan State University, East Lansing, MI 48824 USA

**Keywords:** Gymnotiformes, Hypoxia tolerance, Adaptive evolution, Globin, Positive selection

## Abstract

**Background:**

Nocturnally active gymnotiform weakly electric fish generate electric signals for communication and navigation, which can be energetically taxing. These fish mainly inhabit the Amazon basin, where some species prefer well-oxygenated waters and others live in oxygen-poor, stagnant habitats. The latter species show morphological, physiological, and behavioral adaptations for hypoxia-tolerance. However, there have been no studies of hypoxia tolerance on the molecular level. Globins are classic respiratory proteins. They function principally in oxygen-binding and -delivery in various tissues and organs. Here, we investigate the molecular evolution of alpha and beta hemoglobins, myoglobin, and neuroglobin in 12 gymnotiforms compared with other teleost fish.

**Results:**

The present study identified positively selected sites (PSS) on hemoglobin (Hb) and myoglobin (Mb) genes using different maximum likelihood (ML) methods; some PSS fall in structurally important protein regions. This evidence for the positive selection of globin genes suggests that the adaptive evolution of these genes has helped to enhance the capacity for oxygen storage and transport. Interestingly, a substitution of a Cys at a key site in the obligate air-breathing electric eel (*Electrophorus electricus*) is predicted to enhance oxygen storage of Mb and contribute to NO delivery during hypoxia. A parallel Cys substitution was also noted in an air-breathing African electric fish (*Gymnarchus niloticus*). Moreover, the expected pattern under normoxic conditions of high expression of myoglobin in heart and neuroglobin in the brain in two hypoxia-tolerant species suggests that the main effect of selection on these globin genes is on their sequence rather than their basal expression patterns.

**Conclusion:**

Results indicate a clear signature of positive selection in the globin genes of most hypoxia-tolerant gymnotiform fishes, which are obligate or facultative air breathers. These findings highlight the critical role of globin genes in hypoxia tolerance evolution of Gymnotiform electric fishes.

**Electronic supplementary material:**

The online version of this article (doi:10.1186/s12862-017-0893-3) contains supplementary material, which is available to authorized users.

## Background

Teleosts of the nocturnally-active neotropical clade Gymnotiformes produce and detect weak electric signals for the purposes of electrolocation and communication [1]. Variation in the patterning or frequency of the electric organ discharge (EOD) plays a vital role in electrical communication during behaviors such as aggression, courtship, and mating [2]. EODs are classified as wave- or pulse-type. Wave-type EODs are formed by regularly-emitted pulses where the pulse duration is approximately equal to the inter-pulse interval thereby approximating a sine wave. Pulse-type EODs are emitted irregularly and the EOD pulse duration is short [3]. The order Gymnotiformes comprises six families: Hypopomidae, Rhamphichthyidae and Gymnotidae, which are all pulse-type, and Sternopygidae, Eigenmannidae and Apteronotidae, which are wave-type [4].

In most species EOs are composed of cells, called electrocytes, derived from muscle tissue. The EO is directly controlled by the nervous system, which commands the electrocytes to fire [3]. Electrocytes are large cells capable of generating large ionic currents, especially sodium (Na^+^) currents. In order to restore the ionic gradient for Na^+^ following action potentials, electrocytes have large amounts of the “sodium pump” Na^+^/K^+^ ATPase; this pump uses one molecule of ATP for every three Na^+^ ions pumped back out of the cell. Thus, the generation of electricity requires energy. Physiological studies show that performance-related costs of EOD generation may be surprisingly high, from 10 up to 30% of routine oxygen consumption [5]. Yet, surprisingly, the O_2_ consumption of gymnotiforms does not differ from that of other similarly sized teleosts [6] suggesting that gymnotiforms have adaptations for energy efficiency.

Gymnotiforms have their highest diversity throughout the ecologically varied lowland aquatic habitats of the vast Amazonian floodplains [7]. Although most gymnotiforms inhabit well-oxygenated streams and rivers (including all wave-type and a few pulse-type species), several lineages (only pulse-type species) have additionally radiated within oxygen-poor, stagnant habitats. Furthermore, all species are in danger of being trapped in shrinking, hypoxic pools during the dry season. Thus, hypoxia poses additional physiological challenges, most importantly, a deficiency in oxygen to fuel oxidative metabolism. As a consequence, coping with hypoxia is a potentially daunting task for gymnotiforms.

Different gymnotiform species possess a variety of anatomical and physiological adaptations to cope with these energetic demands including some common to other fish living in hypoxic environments such as large gill surface area [8], the use of aquatic surface respiration (ASR, in which fish swim to the surface and take in water from the topmost heavily oxygenated layer of water over their gills), and various forms of air-breathing [9]. In addition, some species have gymnotiform-specific means of conserving energy such as decreasing EOD amplitude (which lowers Na^+^ influx and, therefore, the amount of ATP needed to power Na^+^/K^+^ ATPase) during the day when they are inactive, upon encountering hypoxic conditions, or when food is scarce [10–12].

Little is known, however, about molecular adaptations for oxygen delivery or usage. Therefore, in this study, we focused on the well-known oxygen carriers, the globins. Globins are the most widespread respiratory proteins, existing in fungi, plants, and animals [13, 14]. Globins are conserved metalloproteins that typically have seven alpha helices that form a pocket containing an oxygen-binding heme [15]. They have been investigated for over a century, and eight globin types have been identified in vertebrates including hemoglobin (Hb), myoglobin (Mb), and neuroglobin (Ngb). Hb and Mb are best known for their respiratory functions, which play critical roles in the maintenance of cellular oxygen supply in support of aerobic metabolism [16, 17].

Hb is a heterotetramer protein composed by two α- and two β-chains, which are encoded by the corresponding alpha (Hba) and beta (Hbb) globin gene family [18]. Moreover, it is responsible for facilitating O_2_ from the respiratory system to the inner organs via the circulatory system [18]. Mb is a compact and highly soluble monomer protein containing one proto-heme, which is involved in the oxygen storage and transportation within heart and skeletal muscle cells [19], and has a higher oxygen affinity than Hb. Ngb is a monomer and an oxygen-carrying protein essentially restricted to neurons [20], which plays a key role in facilitated diffusion and local storage of O_2_. Although its function is still uncertain, there is general agreement that Ngb is associated with mitochondria and thus oxidative metabolism, and serves a neuroprotective role during hypoxic stress [13]. Interestingly, Hb, Mb and Ngb have also been proposed to have roles in nitric oxide (NO) metabolism and the detoxification of reactive oxygen species during hypoxia [21].

We gathered coding sequences of Hba, Hbb, Mb, and Ngb genes from 12 gymnotiform species to: 1) test whether these globin genes have evolved adaptively (i.e.,—show signs of positive selection) during gymnotiform origins and evolution; 2) evaluate whether gymnotiforms that inhabit hypoxic/anoxic vs. well-oxygenated water display different patterns of molecular evolution and; 3) provide a more comprehensive picture of the hypoxia tolerance in gymnotiforms.

## Methods

### Taxon sampling, and primary treatments of data

**Fig. 1:**
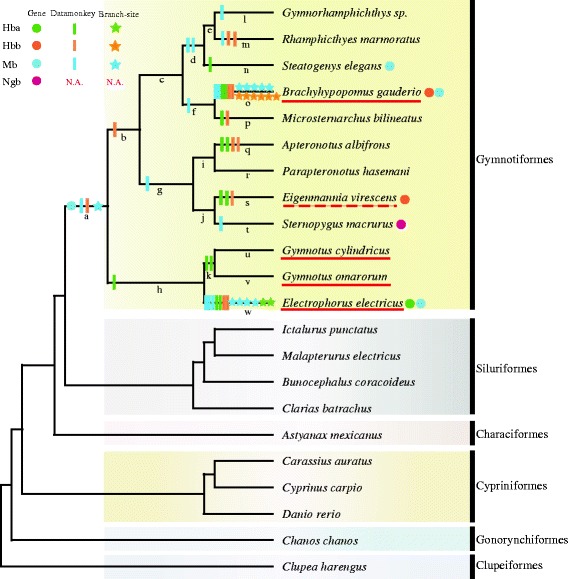
Radical amino acid changes of positively selected sites are shown above the branches across gymnotiformes species tree from a-w. *Circles* indicate positively selected genes across branches. *Bars* represent radical amino acid changes of sites detected by Datamonkey. *Stars* indicate amino acid substitution identified by branch-site model. Species underlined are facultative (or obligate in the case of *E. electricus*) air breathers. *Dotted* species (*E. virescens*) is mildly hypoxia tolerant. Hba, Hbb, Mb and Ngb are colored with *green*, *orange*, *blue* and *pink*, separately

A total of 12 individuals representing six families across Gymnotiformes were analyzed in our study (see Table 1). Some species live in habitats with persistent hypoxia or anoxia while the others are unable to tolerate severely hypoxic water (see Table 1). In order to attain a broad and balanced taxonomic coverage, we also obtained sequences from Siluriformes, Characiformes, Cypriniformes, Gonorynchiformes, and Clupeiformes (see Additional file 1: Table S1). The tree topology used for the analysis is depicted in Fig. 1 [4]. Nucleotide and protein sequences of species were downloaded from National Center for Biotechnology Information (NCBI: http://www.ncbi.nlm.nih.gov/), from NextGen-derived sequences available on a website hosted by the Electric Fish Genome Consortium (http://efishgenomics.zoology.msu.edu/blast), and/or amplified by Polymerase Chain Reaction (PCR). A complete list of non-gymnotiform specimens and accession numbers are in Additional file 1: Table S1.

**Table 1:** Characteristics of species used in this study

Family	Species	Hypoxia-tolerant	Air breather	EOD type	Positive selected genes
Gymnotidae	*Electrophorus electricus*	yes	obligate	Pulse	*Mb, Hba*
Gymnotidae	*Gymnotus cylindricus*	yes	facultative	Pulse	
Gymnotidae	*Gymnotus omarorum*	yes	facultative	Pulse	
Hypopomidae	*Brachyhypopomus gauderio*	yes	facultative	Pulse	*Mb, Hbb*
Hypopomidae	*Microsternarchus bilineatus*	no	no	Pulse	
Hypopomidae	*Steatogenys elegans*	no	no	Pulse	*Mb*
Rhamphichthyidae	*Gymnorhamphichthys sp.*	no	no	Pulse	
Rhamphichthyidae	*Rhamphicthys marmoratus*	no	no	Pulse	
Apteronotidae	*Apteronotus albifrons*	no	no	Wave	
Apteronotidae	*Parapteronotus hasemani*	no	no	Wave	
Sternopygidae	*Eigenmannia virescens*	mildly	no	Wave	*Hbb*
Sternopygidae	*Sternopygus macrurus*	no	no	Wave	*Mb, Ngb*

Note: Designation of hypoxia tolerance based on habitat choice and physiological tests of hypoxia tolerance [10, 11, 72]

### Genomic DNA and tissue RNA isolation and sequencing

Genomic DNA was extracted using QIAGEN DNeasy Tissue Kit (QIAGEN, Inc.). The total RNA was isolated from skeletal muscle, electric organ and brain using the QIAGEN RNeasy Mini Kit (QIAGEN, Inc.), and used as a template for single-stranded (ss) cDNA synthesis using the SuperScript III reverse transcriptase protocol (Invitrogen). Degenerate and specific primers were designed using the homologous sequences from the genome of electric fishes by using Oligo [22]. The primers are listed in Additional file 1: Table S3. This ss cDNA (1:10 dilution) and genomic DNA were used as the template with degenerate primer pairs in the first nested PCR, and their products were templates in second nested PCR reactions with primer pairs. PCR amplifications were carried out using the following program: 95 °C 4 min, 35 cycles of 94 °C denaturation 1 min, 55–50 °C annealing 30 s, 72 °C extension 1 min, and 72 °C elongation for 15 min. PCR products were sequenced in both directions. The newly determined sequences were deposited in GenBank under the accessions KX827275-KX827283, KX833066-KX833070 and KX982253- KX982258.

### Phylogenetic reconstruction

Sequences were assembled and primer sequences were trimmed using MacVector [23]. Nucleotide sequences were translated into amino acid sequences and aligned using MEGA 6.0 [24]. The alignments were manually inspected and edited by eye (see Additional file 2: Figure S1). The best fit model of nucleotide evolution was determined by jModeltest [25], and Maximum Likelihood (ML) trees was reconstructed by PhyML 3.0 [26].

### Molecular evolutionary analysis

To determine whether adaptive evolution might have occurred on the globin genes of electric fishes, we used the PAML package [27], which uses a maximum-likelihood (ML) approach to calculate nonsynonymous to synonymous rate ratios (ω = *dN/dS*). The ratios >1, =1 and <1 indicate positive selection, neutrality and negative selection, respectively. For all genes, both the species tree (Fig. 1) and putative ML tree (Additional file 2: Figure S2) were separately used as the working topology in all the analyses.

We used a site model for positive selection at individual codons in electric fish samples for each gene, i.e., M8 and M8a [28]. Model M8a only allows codons to evolve neutrally or under purifying selection (ω <1), whereas M8 model includes a class of sites with ω > 1. Amino acids under selection for model M8 were identified using a Bayes empirical Bayes approach (BEB), and we considered as candidates sites with a posterior probability >80% [29]. Then, we further employed a series of ML methods implemented in the Datamonkey web server (http://www.datamonkey.org), which has the advantage of improving the estimation of the *dN/dS* ratio by incorporating variation in the rate of synonymous substitution [30]. The single likelihood ancestor counting (SLAC) model is a conservative test, which counts the synonymous and nonsynonymous changes at each codon position in a phylogeny. The Fixed-Effect Likelihood (FEL) calculates site-by-site *dN/dS* without assuming a prior distribution. The Random-Effect Likelihood (REL) assumes a prior distribution across sites. In addition, the Fast Unconstrained Bayesian AppRoximation (FUBAR) ensures robustness against model misspecification. Each module was run using the default cutoffs with *p* = 0.2 for SLAC and FEL, Bayes Factor = 50 for REL and posterior probability = 0.8 for FUBAR.

To test for possible heterogeneity of ω ratios along independent branches, we used the free-ratio model, which allows each branch to have a separate *dN/dS*. The null model is a very strict model called the one-ratio model (M0) that allows only a single ω ratio for all branches. We further executed branch-site tests to explore positive selection affected by a few sites along a specific branch [31]. We compared modified model A, which assumes four classes of sites, especially, allowing codons under positive selection along foreground lineage with ω_2_ > 1, to the null hypothesis, in which fixed ω_2_ = 1 is allowed based on branch-site model A. For all the analyses, the nested models were compared using a likelihood ratio test (LRT) with various degrees of freedom, and all analyses were run twice to ensure convergence. Branch-site REL analysis was also performed to determine whether specific-lineage is evolving under positive selection using a web-based implementation of HyPhy package (http://www.datamonkey.org), which is based on likelihood ratio tests that identify all lineages with a proportion of sites that are evolving with *dN/dS* > 1, and do not require partitioning lineages into foreground and background branches [32].

Each gene sequence alignment was also analyzed using the program TreeSAAP [33], which further supports PAML and Datamonkey at the protein physicochemical level. TreeSAAP compares the magnitude of physicochemical changes of non-synonymous residues across a phylogeny and identifies specific amino acid properties that have likely been affected by positive destabilizing selection during evolution. In this study, amino acid properties were considered to be under positive-destabilizing selection if positive selection was detected in radical magnitude ranges (6–8). The number of radical changes in the amino acid properties was used as a proxy for determining the strength of positive selection. More radical changes in amino acid properties might suggest adaptive evolution. The residues that had fewer than six amino acid property changes were categorized as type I sites, whereas those that had more than six were categorized as type II sites.

### Structural analyses

To provide further insights into the underlying effects of these positively selected sites, we mapped them onto the three-dimensional (3D) structure. The crystal structures of P11748 for Hba, P11749 for Hbb, and 2NRL for Mb were taken from the Protein Data Bank (http://www.rcsb.org/pdb). Postulated functional regions or residues were searched from Uniprot (http://www.uniprot.org/uniprot). Pymol [34] was used to produce the images of the three-dimensional models of corresponding gene.

### Transcriptome assembly

Tissues were removed from a single *Brachyhypopomus gauderio* that was previously housed under normoxic conditions in the laboratory and total RNA was extracted from brain, skeletal muscle, heart and electric organ. RNA was treated with RiboZero (Illumina, MRZH-11124) kit to remove ribosomal RNA, and cDNA libraries (200 bp, paired ends) were made. Libraries were sequenced on an Illumina HiSeq machine. We processed the raw reads with Trimmomatic v0.32 [35] for adapter removal (IlluminaClip: TruSeq3-PE.fa:2:30:10), quality trimming (SlidingWindow:4:5, Leading:5, Trailing:5) and size filtering (MinLen:25). These are Trinity’s default settings, which are based on the work of MacManes [36]. We performed quality control of both raw and processed reads with FastQC v0.11.3 (http://www.bioinformatics.babraham.ac.uk/) (Table S6).

We combined the processed PE reads across organs*, in silico* normalized, and *de novo* assembled them with Trinity v2.2.0 [37, 38]. Then we used BUSCO v1.1b1 [39], along with BLAST+ v2.2.31 [40], HMMER v3.1b1 [41], and EMBOSS v6.5.7 [42] to measure transcriptome completeness, against the Vertebrates, Metazoans and Eukaryotes datasets. In all cases, the assembly displayed a large percentage of complete orthologs (Table S7).

We estimated levels of expression for each transcript and gene, per organ, using the Trinity-provided scripts align_and_estimate_abundance.pl and abundance_estimates_to_matrix.pl. We used both RSEM v1.2.19 [43] and kallisto v0.42.5 [44] quantification methods, which produced qualitatively very similar results. Reported abundances are TMM-normalized values calculated with the RSEM method. Only one Trinity gene blasted against the myoglobin gene sequence with an E-value of 0.00. This gene’s per organ abundances are the ones reported.

The abundance estimation results suggested that very few genes accounted for a large fraction of gene expression. Upon inspection of said genes, many were related with rRNA, and therefore were expected to be depleted during the library preparation process. Although our *B. gauderio* transcriptome assembly meets our quality standards, we don’t recommend future use of the RiboZero kit (which is designed for human, mouse and rat) when working with RNAseq from this taxon. Sequences for *B. gauderio* genes are available at: http://efishgenomics.integrativebiology.msu.edu/blast_search/.

## Results

### Phylogenetic analyses

We successfully amplified cDNAs for the Mb, Hba, Hbb and Ngb gene from eight Gymnotiforms species (see Additional file 1: Table S2). There are no insertion/deletion mutations or changes that result in stop codons in gene sequences, suggesting the presence of functional proteins in electric fishes. These were added to mRNA sequences previously obtained by NextGen sequencing from four other species for a total of 12 species.

We constructed phylogenetic trees using maximum likelihood (ML) performed by PhyML from nucleotide alignments of the four genes dataset. The Akaike Information Criteria (AIC) in jModeltest selected the GTR + G substitution model for all genes. Relationships of the gene trees largely reflected species relationships previously estimated with morphological data by Tagliacollo et al. [4] (see Additional file 2: Figure S2). For example, the phylogenetic tree placed Gymnotiformes and Siluriformes together, and they had a closer relationship with Characiformes than Cypriniformes in the Mb gene tree. However, the topology based on the genes still failed to resolve the relationships within Gymnotiformes, which is not surprising since this has been difficult to resolve even with large datasets. The bootstrap support values for these relationships were not high, which is most likely due to the short length of globin genes and small number of species that were used to reconstruct the topologies.

### Adaptive molecular evolution

Considering that selection analyses using the species tree was basically the same as that using the gene trees, only the former analyses are shown here. First, we used a pair of site models (M8 vs. M8a) to address whether recurrent positive selection has acted on specific codons in globin genes. Likelihood ratio tests (LRTs) showed that a model that includes sites with *dN/dS* >1 (M8) fits the data significantly better than a neutral model (M8a) for the Hbb gene, and one positively selected site was identified with high posterior probability (pp) (Hbb: 133, pp = 0.996) (see Additional file 1: Table S4). Because CodeML does not incorporate rate variation in synonymous sites (d_S_), we therefore further analyzed the data using the SLAC, FEL, REL, and FUBAR model implemented in the Datamonkey website, which have the advantage of improving the estimation of the ω ratio incorporated variation in the rate of synonymous substitution (Pond and Frost 2005a). Similarly, four ML methods also detected sites under selection for Mb, Hba and Hbb genes, some of which coincide with the codons previously identified by M8 (see Table 2). Seven positively selective sites were identified using the SLAC method at significance level of 0.2 (Mb: 2, Hba: 3, Hbb: 2). The FEL methods found 17 positively selected codons (Mb: 4, Hba: 5, Hbb: 8). Using the REL method, 8 codons were identified under positive selection at significant level of Bayes factor > 50 (Mb: 2, Hba: 4, Hbb: 2). Furthermore, FUBAR also identified 14 sites under diversifying selection with a posterior probability > 0.8 (Mb: 4, Hba: 4, Hbb: 6) (see Table 2). It is generally accepted that a positively selected site is more reliable if it can be supported by two or more different methods. Of these putative positively selected sites, 13 codons (Mb: 27, 92, 98; Hba: 35, 73, 79, 83, 109; Hbb: 15, 81, 123, 133, 144) were detected by more than one ML methods, which are robust candidates for sites under selection; especially, site 133 in Hbb was determined to have undergone positive selection by all methods (see Table 2).

**Table 2:** Evidence of positive selection identified by different methods in Hyphy

Gene	SLAC (P > 0.8)	FEL (P > 0.8)	REL (PP > 50)	Fubar (PP > 0.8)	Branch-site REL
*Hba*	35 (0.134), 73 (0.199), 109 (0.133)	35 (0.058), 73 (0.077), 79 (0.111), 83 (0.132), 109 (0.073)	35 (60.944), 73 (60.689), 83 (50.668), 109 (84.333)	35 (0.909), 73 (0.840), 79 (0.940), 109 (0.824)	*E.electricus*: p = 0.007
*Hbb*	123 (0.197), **133** (0.154)	15 (0.093), 45 (0.146), 81 (0.127), 106 (0.164), 112 (0.162), 123 (0.072), **133** (0.067), 144 (0.100)	123 (60.003), **133** (1832.56)	15 (0.818), 81 (0.874), 111 (0.899), 123 (0.854), **133** (0.979), 144 (0.921)	*B.gauderio*: p = 0.003; *E.virescens*: p = 0.037
*Mb*	27 (0.097), 98 (0.096)	27 (0.021), 92 (0.110), 98 (0.029), 108 (0.131)	27 (337.517), 98 (94.269)	27 (0.973), 44 (0.849), 92 (0.849), 98 (0.925)	*E.electricus*: p = 0.005 *B.gauderio*: p = 0.017

Note: Codons identified by more than one ML method are underlined. Sites also detected by M8 model are shown in bold. Site positions were relative to Zebrafish (*Danio rerio*) protein sequences, i.e., Mb (Q6VN46), Hba (Q90487), Hbb (Q90486), and Ngb (Q90YJ2) in UniProtKB

To test for positive selection along particular branches, we used the one-ratio model (M0) that allows only a single ω ratio for all branches. The ω ratio estimated by M0 is significantly less than 1 (from 0.075 to 0.296) (see Additional file 1: Table S5), suggesting that strong purifying selection plays a central role in the evolution of globin genes in electric fishes. The codeml-free ratio branch model of PAML estimated independent ω along all branches of the phylogeny, which was significantly better than the one-ratio model (p < 0.001, see Additional file 1: Table S5) for Mb, Hba and Hbb, suggesting heterogeneous selective pressures on different lineages. To investigate whether the evidence for positive selection was restricted to individual codons along some specific lineages, branch-site models were then performed. Foreground branches for tests of positive selection were selected on clades of Gymnotiforms. The LRT tests showed that four Mb branches, one Hba branch, one Hbb branches and one Ngb branch exhibited significant (P < 0.05) evidence of positive selection over background branches, separately (see Table 3). Further evidence of positive selection was also detected by Branch-site REL. The results show that lineages leading to *Brachyhypopomus gauderio* (branch o, p = 0.017) and *Electrophorus electricus* (branch w, p = 0.005) were under positive selection for the Mb gene. Additionally, this model also confirms episodic selection along the same lineages (Mb: branch o, p = 0.017 and branch w, p = 0.005; Hba: branch w, p = 0.007; Hbb: branch o, p = 0.003) reported by the branch-site model in PAML, but also identifies an additional lineage—the *Eigenmannia virescens* (branch s, p = 0.037) lineage for Hbb gene (see Table 2). To summarize the above results from the analyses using different methods, all four globin genes were identified as having undergone positive selection in Gymnotiforms.

**Table 3:** Selective pressure analyses of Gymnitifores by the Branch-Site Model

Gene	Branch	Model	Ln	2ΔLn	*p*	Parameter	Positive selected sites
*Hba*	*E.electricus*	Ma	3039.610			ω0 = 0.129, ω1 = 1, ω2 = 6.251	18-0.995**, 46–0.990*
		Ma0	3042.305	5.390	0.020	ω0 = 0.129, ω1 = 1, ω2 = 1	
*Hbb*	*B.gauderio*	Ma	3177.724			ω0 = 0.145, ω1 = 1, ω2 = 34.694	20-0.955*, 29–0.973*, 113–0.989*, 114–0.912, 132–0.981*, 133–0.888
		Ma0	3187.339	19.230	<0.001	ω0 = 0.139, ω1 = 1, ω2 = 1	
*Mb*	*B.gauderio*	Ma	5191.675			ω0 = 0.137, ω1 = 1, ω2 = 15.025	9-0.987*, 18–0.964*, 98–0.940, 109–0.990**, 122–0.994**, 134–0.992**, 138–0.911
		Ma0	5199.405	15.46	<0.001	ω0 = 0.136, ω1 = 1, ω2 = 1	
	*E.electricus*	Ma	5191.521			ω0 = 0.136, ω1 = 1, ω2 = 381.189	37-0.998**, 106–0.982*, 108–0.960*, 109–0.993**, 117–0.944
		Ma0	5195.127	7.212	0.007	ω0 = 0.132, ω1 = 1, ω2 = 1	
	*S.elegans*	Ma	5201.909			ω0 = 0.142, ω1 = 1, ω2 = 999	38-0.933
		Ma0	5204.687	5.556	0.018	ω0 = 0.141, ω1 = 1, ω2 = 1	
	Last common ancestor of gymnotiform	Ma	5199.339			ω0 = 0.143, ω1 = 1, ω2 = 60.065	12-0.986*
		Ma0	5204.040	9.402	0.002	ω0 = 0.141, ω1 = 1, ω2 = 1	
*Ngb*	*S.macrurus*	Ma	2223.853			ω0 = 0.063, ω1 = 1, ω2 = 189.298	150-0.989*
		Ma0	2226.360	5.014	0.025	ω0 = 0.063, ω1 = 1, ω2 = 1	

Note: *: posterior probability (pp) >95%; **: pp >99%

Although Codeml and Datamonkey estimate the influence of natural selection at the codon level, selection for change in amino acid physicochemical properties was also analyzed by TreeSAAP, which further support the ML methods results at a complementary protein-level. Overall, 33 residues in four globin genes were subjected to positive selection supported by two or more ML methods (Datamonkey, site model and Branch-site model), and 78.8% (26/33) (Hba: 6, Hbb: 9, Mb: 11) were also detected by TreeSAAP as under positive selection at the physicochemical level (see Table 4). Nine of these sites were Type II class (more than or equal to six radical changes in amino acid properties). Furthermore, the radical amino acid changes in the 26 positively selected codons were scattered throughout most of the Gymnotiform phylogeny (see Fig. 1). It is interesting to note that high levels of positive selection were found in “hypoxic water” species compared with “well-oxygenated water” species; that is, 15 radical amino acid sites (Hba: 2, Hbb: 7, Mb: 6) were detected in branch o; 13 radical amino acid sites (Hba: 4, Hbb: 2, Mb: 7) were identified in branch w; and 3 radical amino acid sites (Hba: 2, Hbb: 1) were found in branch s (see Fig. 1).

**Table 4:** Radical amino acid sites under positive selection detected by Datamonkey, Branch-site model and TreeSAAP Simultaneously

Gene	Position^a^	Clade^b^	AA Changes	Radical Changes in AA Properties^c^	Total
*Hba*	18	w	I-A	*pK’*, *R*_*a*_,	2
	35	k	T-A	*P*_*α*_	1
		s	T-V	*N*_*s*_, *R*_*a*_	2
		n	T-P	*K* ^*o*^, *H*_*t*_	2
		o	T-I	***N***_***s***_ **,** ***B***_***r***_ **,** ***pK’*** **,** ***R***_***a***_ **,** ***H***_***p***_ **,** ***H***_***t***_	**6**
	46	w	H-S	*F*, *E*_*t*_	2
	73	h, s	S-A	*P*_*α*_, *P*_*c*_, *P*	3
		w	A-G	*P*_*α*_, *P*_*c*_, *P*	3
	79	o, w	A-T	*P*_*α*_	1
		p, q	A-C	***P***_***α***_ **,** ***N***_***s***_ **,** ***c*** **,** ***K*** ^***o***^ **,** ***pK’*** **,** ***α***_***n***_	**6**
	83	k, q	A-S	*P*_*α*_, *P*_*c*_, *P*	3
*Hbb*	15	p	M-I	*pK’*	1
	20	o	S-H	*F*	1
	29	o	L-I	*pK’*	1
	81	s	N-A	*P*_*α*_, *P*_*c*_, *P*	3
	113	o	V-T	*N*_*s*_, *R*_*a*_	2
	114	o	C-T	*N*_*s*_, *B*_*r*_, *c*	3
	123	m, w	V-G	***P***_***β***_ **,** ***B***_***l***_ **,** ***P***_***c***_ **,** ***F*** **,** ***R***_***a***_ **,** ***P***	6
		o, q	V-A	*P*_*β*_	1
	132	o	W-I	*pK’*	1
	133	a	Q-C	***N***_***s***_ **,** ***B***_***r***_ **,** ***c*** **,** ***h*** **,** ***p*** **,** ***E***_***t***_	6
		h	C-S	*N*_*s*_, *B*_*r*_, *μ*	3
		w	S-A	*P*_*α*_, *P*_*c*_, *P*	3
		q	C-Q	***N***_***s***_ **,** ***B***_***r***_ **,** ***c*** **,** ***h*** **,** ***p*** **,** ***E***_***t***_	6
		m	C-T	*N*_*s*_, *B*_*r*_, *c*	3
		o	C-E	***P***_***α***_ **,** ***N***_***s***_ **,** ***B***_***r***_ **,** ***c*** **,** ***h*** **,** ***E***_***l***_ **,** ***P***_***r***_ **,** ***p*** **,** ***α***_***c***_ **,** ***H***_***p***_ **,** ***E***_***t***_	11
*Mb*	9	o	K-A	*E*_*t*_, *B*_*r*_, *K* ^*o*^, *h*, *E*_*sm*_	5
	12	a	G-P	*B*_*l*_, *α*_*c*_, *H*_*t*_	3
	27	w	T-G	*B*_*l*_,	1
		l	T-V	*N*_*s*_, *R*_*a*_	2
		o	S-W	***B***_***l***_ **,** ***R***_***F***_ **,** ***P***_***c***_ **,** ***C***_***a***_ **,** ***E***_***l***_ **,** ***F*** **,** ***M***_***v***_ **,** ***M***_***w***_ **,** ***V*** ^**0**^ **,** ***u*** **,** ***R***_***a***_ **,** ***H***_***t***_	**12**
	37	w	Q-I	***N***_***s***_ **,** ***B***_***r***_ **,** ***pK’*** **,** ***h*** **,** ***F*** **,** ***p*** **,** ***E***_***t***_ **,** ***E***_***l***_ **,** ***R***_***a***_ **,** ***H***_***p***_ **,** ***H***_***t***_	**11**
	92	w, f	K-Q	*Ph*_*i*_	1
		g	K-I	***N***_***s***_ **,** ***P***_***β***_ **,** ***B***_***r***_ **,** ***R***_***F***_ **,** ***pK’*** **,** ***h*** **,** ***E***_***l***_ **,** ***F*** **,** ***H***_***nc***_ **,** ***P***_***r***_ **,** ***p*** **,** ***R***_***a***_ **,** ***H***_***p***_ **,** ***E***_***t***_	**14**
		t	I-V	*pK’*	1
		d	K-T	*Ph*_*i*_, *E*_*t*_	2
	98	a	N-A	*P*_*α*_, *P*_*c*_, *P*	3
		w	A-I	*pK’*, *R*_*a*_	2
		d	A-G	*P*_*α*_, *P*_*c*_, *P*	3
		m	G-V	***P***_***β***_ **,** ***B***_***l***_ **,** ***P***_***c***_ **,** ***F*** **,** ***R***_***a***_ **,** ***P***	**6**
		o	A-N	*P*_*α*_, *P*_*c*_, *P*	3
	106	w	V-C	*pK’*, *C*, *R*_*a*_, *P*	4
	108	w	V-C	*pK’*, *C*, *R*_*a*_, *P*	4
	109	w	K-V	***N***_***s***_ **,** ***B***_***r***_ **,** ***h*** **,** ***E***_***l***_ **,** ***F*** **,** ***p*** **,** ***R***_***a***_ **,** ***H***_***p***_ **,** ***H***_***nc***_ **,** ***E***_***t***_	**10**
		o	K-S	*E*_*t*_	1
	122	o	Q-A	*K* ^*o*^, *E*_*t*_	2
	134	o	N-A	*P*_*α*_, *P*_*c*_, *P*	3

^a^Relative to Zebrafish (*Danio rerio*) protein sequences, i.e., Mb (Q6VN46), Hba (Q90487), and Hbb (Q90486) in UniProtKB. Codons identified as under positive selection more than one ML methods. Positively selected sites detected by Datamonkey are represented in underlined letters

^b^Amino acid substitution occurred along clades, with detailed information marked in Fig. 1

^c^Radical changes in amino acid properties under category 6–8 were detected in TreeSAAP. Amino acid sites that belong to the Type II class (greater than 6 property changes) are represented in bold. Physicochemical amino acid properties available in TreeSAAP are as following: *α*_*c*_: Power to be - C-term., α-helix; *α*_*n*_: Power to be in the N-terminal of an α-helix; *B*_*r*_: Buriedness; *c*: Composition; *C*_*a*_: Helical contact energy; *E*_*l*_: Long-range non-bonded energy; *E*_*sm*_: Short and medium range non-bonded energy; *E*_*t*_: Total non-bonding Energy; *F*: Mean r.m.s. fluctuation displacement; *h*: Hydropathy; *H*_*nc*_: Normal consensus hydrophobicity; *H*_*p*_: Surrounding hydrophobicity; *H*_*t*_: Thermodynamic transfer hydrophobicity; *K* ^*o*^: Compressibility; *μ*: Refractive index; *M*_*v*_: Molecular volume; *M*_*w*_: Molecular weight; *N*_*s*_: Average number of surrounding residues; *P*_*α*_: α- helical tendencies; *P*_*β*_: β-structure tendencies; *P*_*c*_: Coil tendencies; *P*: Turn tendencies; *p*: Polarity; *pH*_*i*_: Isoelectric point; *pK’*: Equilibrium Constant of ionization for COOH; *P*_*r*_: Polar requirement; *R*_*a*_: Solvent accessible reduction ratio; *R*_*F*_: Chromatographic index; *V* ^0^: Partial specific volume

### Structural links to protein function

**Fig. 2:**
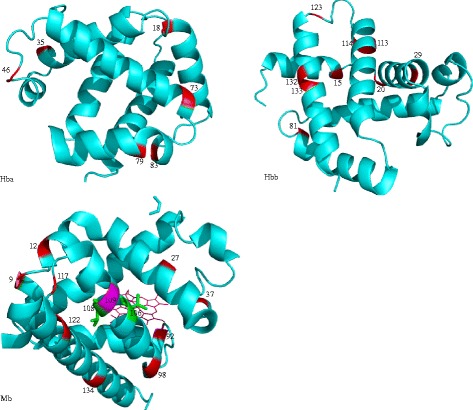
Radical changes of sites under selection are shown in crystal structure of a: Hba, b: Hbb, and c: Mb with *red*. The site in Mb identified along lineages leading to *Brachyhypopomus gauderio* and *Electrophorus electricus* is marked with *pink* (residue 109). Cys substitutions of *Electrophorus electricus* at Mb gene are marked with *green* (residue 106 and 108)

To gain insight into the functional significance of the putatively selected sites, we mapped all the radical amino acid sites onto 3D structures. We found that several sites fall in or immediately adjacent to the functional regions or residues (see Fig. 2). For example, site 83 in Hba gene was close to the heme proximal ligand residue (site 89 in Zebrafish). Again, site 144 in Hbb was adjacent to the β-147 histidine. For the Mb gene, sites 9, 12, 27, 37 and 38 were located in the globin region. 42.3% (11/26) positively selected sites with radical changes were localized in residues postulated to affect function.

### Expression profile of Mb and Ngb in *E. Electricus*

**Fig. 3:**
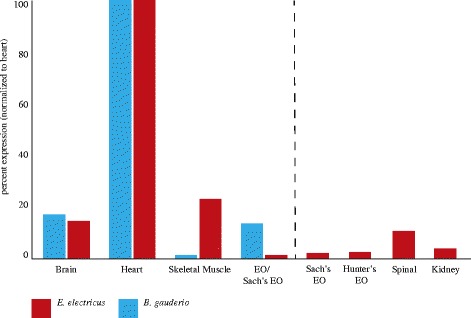
Tissue distribution of myoglobin of the electric eel (*Electrophorus electricus*) and the pintail knifefish (*Brachyhypopomus gauderio*)

**Fig. 4:**
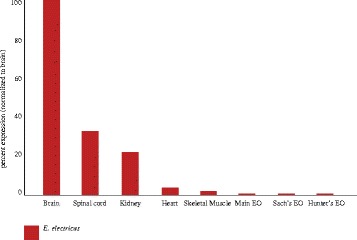
Tissue distribution of neuroglobin of the electric eel (*Electrophorus electricus*)

We wished to compare expression patterns under normoxic conditions of *E. electricus* and *B. gauderio* Mb and Ngb with other teleosts. We did not measure levels of hemoglobins in these tissues because hemoglobins are only expressed in erythrocytes; any signal from hemoglobins would be from residual red cells left in the tissues rather than expression in the tissues themselves. As a result of earlier work [45, 46], we had access to tissue transcriptomes of *E. electricus* and examined levels of Mb and Ngb expression (see Fig. 3). Mb is highly expressed in the heart and less so in muscle. Mb is also expressed in some non-muscle-derived tissues such as brain, spinal cord, and kidney. Surprisingly, it is only negligibly expressed in the EOs (*E. electricus* has two weak EOs for communication and navigation and one strong EO for shocking prey). Ngb is expressed in the brain, spinal cord, and kidney, and at negligible levels elsewhere (see Fig. 4).

Transcriptomes were made from some of the same tissues (brain, heart, skeletal muscle, EO) of *B. gauderio* (see Additional file 1: Table S6 and S7; Additional file 3). The overall pattern of Mb expression is similar to *E. electricus*. That is, expression is highest in the heart, also observed in brain, and low in muscle and EO. Ngb levels were too low to measure accurately.

## Discussion

### Pervasive adaptive evolution of gymnotiform globin genes

Recent studies have shown strong evidence for positive selection in hypoxia tolerant species, like yak [47], hummingbirds [48], and cetaceans [49], when compared with their hypoxia intolerant relatives, as well as hypoxia-tolerant populations of humans such as Tibetans [50].

In this study, we surveyed all four primary oxygen-carrier globin genes in gymnotiforms for signs of positive selection and to assess whether hypoxia tolerance has influenced the evolution of these genes. Our analyses provide strong evidence that globin genes have been subjected to positive selection during gymnotiform evolution. First, neutral models of evolution were rejected for Hbb genes, and more than two ML methods identified specific codons with a high probability of being under selection for Hba, Hbb and Mb genes. Second, adaptive evolution was further supported by evidence of radical changes in positively selected amino acids in gymnotiform globins. Again, 34.6% (9/26) belong to the Type II class, suggesting robust positive selection. Finally, several of the putatively selected sites fall in, or close to, regions important for function based on structural information.

Positively selected sites are scattered pervasively along lineages of gymnotiform phylogeny (see Table 2 and Fig. 1), suggesting the contribution of the respiratory proteins for oxygen storage and transportation during adaptation to expensive oxygen consumption in gymnotiforms. Moreover, a signal of positive selection was also detected in the lineage leading to the common ancestor of gymnotiforms. This lineage represents the early evolutionary history of the gymnotiform’s evolved EOs, during which the gymnotiforms were faced with the challenges of high energetic cost for electric organ discharge generation. Although this branch was only detected in the Mb gene with three positively selected codons (Mb: N98A, G12P; Hbb: Q133C), three or more radical property changes occurred at each amino acid (see Table 4). That is to say, globin genes may have adaptively enhanced oxygen binding and transportation in accordance with the changes of high energetic cost during the early evolutionary phase of EOs in gymnotiformes.

### Functional consequences of amino acid replacement

Although gymnotiform globin genes contain putatively positively selected sites, it is important to assess their functional relevance. We thus analyzed selected residues for their structural properties to predict potential functional implications. We found that all 26 radical amino acid changes residues were concordant between three methods and thus constitute robust candidates for positive selection (see Table 4). Radical substitutions of amino acids at key positions may change the properties of the molecule. For example, residue 83 in Hba is located very close to the promixal histidine, which is implicated in the iron-proximal histidine linkage [51]. Consequently, substitution at this site seems to be essential in the maintenance of heme oxygen binding.

Furthermore, position 144 in Hbb is close to 147 His. It has been reported that His-HC3(147)β carp (*Cyprinus carpio*) hemoglobin plays a key role in the Root effect, which is a phenomenon associated with non-cooperative oxygen binding and decreased oxygen affinity [52]. Hence, we suggest that positive selection acting on this site is likely to be involved in modulating hemoglobin oxygen combination and cooperation. For Mb, site 134 is mainly responsible for formation of hydrogen bonds with water in a hydrophobic environment [53]; therefore, amino acid changes at this site are likely to be involved in the regulation of water bonds. In spite of the evidence for selection documented here, functional studies of these candidate positively selected sites are necessary in gymnotiforms in the future.

### Myoglobin and NO production

**Fig. 5:**
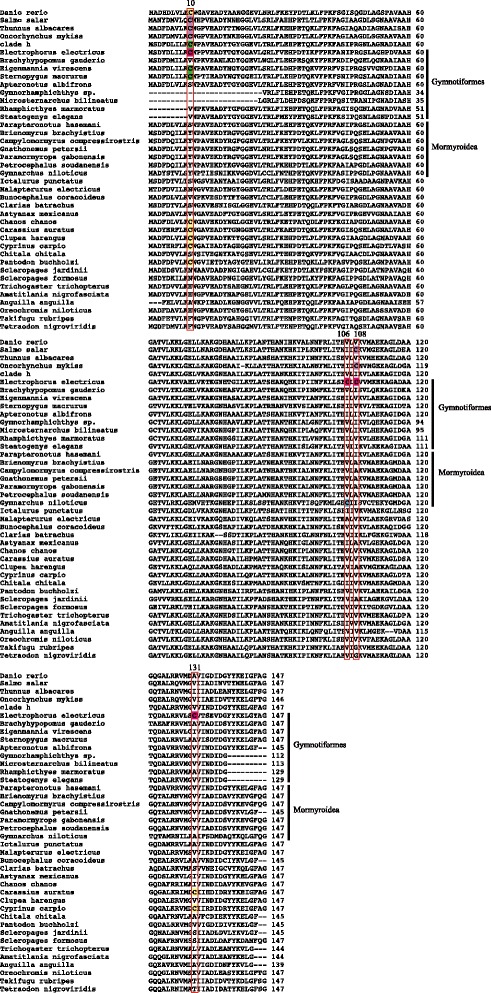
Multiple sequences alignment of 40 teleost Mb gene, showing Cys residues (i.e., 10, 106, 108, and 131) of *Electrophorus electricus* (*pink*). The common substitutions identified are located at the amino acid position of the zebrafish (*Danio rerio*) ortholog. The consensus numbering of zebrafish is given in the right. The Cys 10 and 108 residues of *Salmo salar* and *Thunnus albacares* are shadowed by purple. The corresponding Cys sites in *Gymnarchus niloticus* (Mormyroidea) are shown in *blue*, in Gymnotiformes in *green*, in the remaining species in *orange*. The alignments of each Cys sites are in the *red boxes*. Clade h (Fig. 1) is the common ancestor of the genus *Gymnotus* and *Electrophorus electricus*. NCBI accession numbers are provided in Additional file 1: Table S1

Mb plays a pivotal role in the response to hypoxia. On the one hand, Mb facilitates O_2_ diffusion from the blood into skeletal and heart muscle of vertebrates; on the other hand, deoxy-Mb may act as a nitrite reductase producing NO from NO_2_ in response to cellular hypoxia [13]. Recent studies reported that cysteine (Cys) S-nitrosation of trout and salmon Mb increases heme O_2_ affinity, and this allosteric effect may promote hypoxia-induced NO delivery in the heart and improve myocardial efficiency [54, 55]. Interestingly, *Electrophorus electricus* also contains four reactive cysteine (Cys) residues (see Fig. 5). Cys 10 and 108 are identical to trout and salmon, and Cys 106 and 131 are radical amino acid changes from ancestral Val to Cys (V106C, V131C). Moreover, Cys 106 is also a species-specific site in *E. electricus*, and Cys 108 is identified as a positively selected site. It is worth noting that S-nitrosation at reactive cysteines is functionally indispensible for generating S-nitroso Mb (Mb-SNO) and contributing further to NO homeostasis. Hence, it is reasonable to deduce that reactive Cys sites in *E. electricus* may enhance Mb function in oxygen storage and NO delivery during hypoxia.

Interestingly, the Mb of *Gymnarchus niloticus* a member of the other independently evolved group of electric fishes, the mormyroidea, shares Cys 106 (see Fig. 5, blue) with *E. electricus* at a site that is conserved among other teleosts. Whereas all other (200+ species) of mormyroid fishes utilize gills and cannot breathe air under hypoxic conditions [56–58], *G. niloticus* is an air-breathing fish with a highly vascularized gas bladder. These fish also grow to be large (>1.5 meters) similar to *E. electricus*. Therefore, it appears that the shared Cys 106 is a functional convergence in two large, air-breathing electric fish.

### Relationship of globin evolution to gymnotiform life histories

The two species with strongest signature of positive selection of globins—*E. electricus* and *B. gauderio*--live comfortably in hypoxic and even anoxic environments*. E. electricus,* which is the largest gymnotiform capable of reaching ~2 m in length*,* is unusual among gymnotiforms as it is an obligate air-breather. It surfaces every few minutes to gulp air, which it stores in its mouth and, if prevented from breathing air, it will drown [59]. *E. electricus* is unique even among air breathing fishes as it obtains oxygen from elaborated papillae in the mouth not related to the gills; indeed, its gills are small and underdeveloped. Furthermore, its circulatory system is unlike that of other teleosts in that the oxygenated blood from the oral papillae mixes with the venous circulation before being pumped out of the heart resulting in poorly oxygenated blood. The hematocrit, Hb content, and oxygen capacity of the blood, as well as heart rate and volume of blood moved per unit time are all higher than in most teleosts and may be adaptations to overcome the poor oxygenation of mixed arterial-venous blood [59].

Fish of the genus *Brachyhypopomus* inhabit hypoxic/anoxic waters [10] and in the laboratory, tolerate >6 h of anoxia. They have large well-developed gills, but are also facultative air-breathers either gulping bubbles of air at the surface then descending, or “skulking” at the surface with open mouths taking in air [8]. These two behaviors differ from ASR in that they involve taking in air, whereas ASR is merely taking in well-oxygenated water from the surface layer. Fish that hold air in their mouths must have gills that do not collapse or they would provide too little useful surface area for oxygen absorption.

*Gymnotus* species are also capable of tolerating >6 h of anoxia by gulping air and storing it in a highly vascularized, extended, lung-like posterior swim bladder unique among gymnotiforms [60]. They use this swim bladder “lung” to extend their aerobic range [61]. The swim bladder is contacted by branches of the celiac artery and hepatic portal systems as well as segmental arteries and veins that then penetrate the body musculature. Although we tried multiple primers to amplify *Gymnotus omarorum* Mb from muscle, heart and EO tissue, we failed to amplify this gene from RNA samples even though we obtained the Hba and Hbb genes from the same samples. Recently, Macqueen et al. [62] revealed that a cardiac Mb deficit has evolved repeatedly in teleosts under diverse ecological settings; in sticklebacks (*Gasterosteus aculeuatus*) Mb is even a pseudogene. Although we have not examined *Gymnotus* DNA for a possible psuedogene, we suspect that the Mb gene was lost in *G. omarorum*. In keeping with this, we did not note strong selection on Hbs in this genus. We suggest that their blood is adequately oxygenated in their unique vascularized swim bladder with no further selection pressure to alter Hb affinity for oxygen.

Crampton [10] included *E. virescens* among the species that are capable of inhabiting hypoxic environments. However, this species is just barely able to survive in hypoxia. They tend to avoid hypoxic water although they are capable of surviving an hour or so of anoxic conditions using ASR [10], but they do not gulp air. They are sensitive to hypoxia under which the amplitude of their EODs decreases rapidly [11]. In accordance with this, we observe only minimal evidence of positive selection on Hbb in this species.

The other species in this study, which show little or only weak evidence of positive selection on globin genes, do poorly in hypoxic conditions and cannot survive anoxia [10]. Under anoxic conditions in the laboratory they respond with ASR as long as they are able and eventually fall immobile to the bottom. These include all the wave species and some of the pulse-type fish.

Finally, the genera *Brachyhypopomus* and *Gymnotus* are speciose [4] with member species distributed throughout the gymnotiform’s whole range. For example, fish from these two genera are the primary species in the most Southern extent of the gymnotiform’s distribution in Uruguay [63, 64]. The evolution of different modes of air-breathing may have given them an advantage over those species unable to derive oxygen from the air.

### Expression of Mb and Ngb in *E. electricus* and *B. gauderio*

As in some other teleosts, Mb is most highly expressed in heart (although this varies considerably across species [62]) and less so in muscle. However, as Mb is expressed in “slow” oxidative but not “fast” non-oxidative muscle fibers, and slow fibers are small and few in number in teleost muscle, its mRNA will not be abundant in a sample of epaxial muscle even if it is highly expressed in slow muscle [65, 66]. Thus, the difference in muscle Mb expression between *E. electricus* and *B. gauderio* may depend on composition of the muscle sub-types and the exact location from which samples were obtained.

Our expectation was that Mb levels would be high in the EO consistent with the ongoing EOD activity. But Mb levels are modest in the EO of both species. We did not note a difference in expression between the Sach’s organ and Hunter’s/Main EO of *E. electricus*. Sach’s EO constantly fires a low voltage-pulse at a low frequency (~1–10 Hz), whereas all three EOs discharge in high frequency bursts of >400 Hz when fish are capturing prey or defending themselves [67]. *B. gauderio* discharges at rest at ~15 Hz and during social interactions or foraging at 30–40 Hz, with occasional high-frequency bursts (100–200 Hz) called “chirps” that are aggressive signals [68].

There are a few possible and not mutually exclusive explanations for the low levels of Mb in the EO. First, EO originates from fast muscle fibers, and fast muscle fibers do not express Mb; there might be a developmental constraint minimizing Mb expression in the EO. Second, some gymnotiforms possess mechanisms to decrease EOD amplitude (and therefore oxygen consumption) during anoxia [11]. This has only been studied in a few species and it would be intriguing to compare the distribution of EO Mb expression, EOD “usage” (discharge rate), and occurrence of mechanisms for reducing EOD amplitude in the face of anoxia in the EO across gymnotiforms. Perhaps different species trade off EO Mb concentration with extent of anoxia-dependent reduction of the EOD amplitude. Third, EOs are well vascularized and it may not be necessary to have a local reserve source of O_2_. Fourth, it is possible that EOs switch to anaerobic metabolism during and after periods of high activity. At this point there is no evidence for or against this. It is also worth noting that Mb expression is high in heart and low in muscle in the elephant shark suggesting that this is the ancestral vertebrate pattern [69].

Recently, it has been recognized that Mb is expressed in the brain and kidney as well as in a few other non-muscle tissues in teleosts. Mainly, Mb is expressed in the endothelial cells of blood vessels in the brain and gills, and the epithelial cells of the tubules in the kidney [65]. The expression of Mb in the brain in *E. electricus* and *B. gauderio* and the kidney of *E. electricus* is in accord with these observations.

Ngb is primarily expressed in neural tissues in mammals and teleosts. It is reported to be in the gills in zebrafish [70]. As there are no gill transcriptomes available of *E. electricus* we do not know if it is expressed in the gills of this species. Ngb was expressed in *E. electricus* kidney but zebrafish kidney has not yet been examined. Finally, it is not expressed in zebrafish muscle nor did we observe it in *E. electricus* muscle. Qualitatively, the expression pattern of Mb and Ngb does not differ from non-gymnotiforms. Thus, it appears that the main effect of selection on *E. electricus* and *B. gauderio* Mb is on its sequence rather than its expression pattern in individuals housed under normoxic conditions. Further experiments would be necessary to determine whether there are additional adaptations on the regulation of globin genes under different environmental conditions.

## Conclusion

The gymnotiforms arose in Gondwana [71] and diverged rapidly into lineages with differences in EO morphology and signaling. Some amino acid substitutions occurred in myoglobin genes as the EO was initially evolving, perhaps as a result of the new energy demands imposed by electrogenesis. While many gymnotiforms rely on gills for oxygen uptake, a few lineages evolved distinct modes of air-breathing. Along with this, we note variation in the strength of selection on different globin genes depending on the mode of air breathing. We anticipate that future studies will elucidate the physiology and biochemistry of these globin genes and seek other molecules under selection in the oxygen transport or metabolic pathways underlying energy usage.

## Additional files

Additional file 1: Table S1. List of Species Used in this Study. **Table S2.** Statistics for amplified genes for each species. **Table S3.** List of primers used to amplify the coding regions of globin genes in this study. **Table S4.** Positive Selection Detected by Site Models in PAML. **Table S5.** Positive Selection Detected by Branch Models in PAML. **Table S6.** Raw and Processed Reads from Each Tissue. **Table S7.** Evaluation of transcriptome completeness against the Vertebrates, Metazoans and Eukaryotes datasets. (DOCX 42 kb)
Additional file 2: Figure S1. Sequences alignment of globin genes. **Figure S2** The phylogenetic trees constructed from nucleotide sequences used maximum likelihood tests. (ZIP 729 kb)
Additional file 3: Further details of *Brachyhypopomus gauderio* transcriptome. (DOCX 133 kb)
