# Supplementary material for: Molecular evolution of globin genes in Gymnotiform electric fishes: relation to hypoxia tolerance
Source: BMC Evol Biol. 2017 Feb 13;17:51. doi: 10.1186/s12862-017-0893-3 (PMC5307702; doi:10.1186/s12862-017-0893-3)
Supplement: Additional file 1: Table S1. — List of Species Used in this Study. Table S2. Statistics for amplified genes for each species. Table S3. List of primers used to amplify the coding regions of globin genes in this study. Table S4. Positive Selection Detected by Site Models in PAML. Table S5. Positive Selection Detected by Branch Models in PAML. Table S6. Raw and Processed Reads from Each Tissue. Table S7. Evaluation of transcriptome completeness against the Vertebrates, Metazoans and Eukaryotes datasets. (DOCX 42 kb) [file 12862_2017_893_MOESM1_ESM.docx]

**Supplementary tables:**

Table S1 Accession numbers of Species Used in this Study. - in any genes indicate fail to download from genome. Note: species only used in Mb alignment in figure 5 were labeled in blue.

| Order | Family | Species | *Hba* | *Hbb* | *Ngb* |
| --- | --- | --- | --- | --- | --- |
| Characiformes | Characidae | *Astyanax mexicanus* | XM_007238189.1 | XM_007238187.2 | XM_007232642.1 |
| Cypriniformes | Cyprinidae | *Danio rerio* | BC164447.1 | NM_131020.2 | AJ315610.1 |
|  | Cyprinidae | *Carassius auratus* | AM933143.1 | AM933144.1 | AM933145.1 |
|  | Cyprinidae | *Cyprinus carpio* | AF528156.1 | D88115.1 | KC342291.1 |
| Clupeiformes | Clupeidae | *Clupea harengus* | XM_012833788.1 | XM_012833783.1 | - |
| Siluriformes | Ictaluridae | *Ictalurus punctatus* | NM_001201272.1 | KF471108.1 | XM_017475501.1 |

| Order | Family | Species | *Mb* |
| --- | --- | --- | --- |
| Characiformes | Characidae | *Astyanax mexicanus* | XM_007229538.1 |
| Cypriniformes | Cyprinidae | *Danio rerio* | NM_200586.1 |
|  | Cyprinidae | *Carassius auratus* | AM747267.2 |
|  | Cyprinidae | *Cyprinus carpio* | KC342292.1 |
| Clupeiformes | Clupeidae | *Clupea harengus* | XM_012836002.1 |
| Gonorynchiformes | Chanidae | *Chanos chanos* | AB480168.1 |
| Siluriformes | Aspredinidae | *Bunocephalus coracoideus* | KJ561855.1 |
|  | Clariidae | *Clarias batrachus* | KC206067.1 |
|  | Ictaluridae | *Ictalurus punctatus* | NM_001200597.1 |
| Anabantiformes | Osphronemidae | *Trichogaster trichopterus* | KJ561852.1 |
| Anguilliformes | Anguillidae | *Anguilla anguilla* | KJ561856.1 |
| Cichliformes | Cichlidae | *Amatitlania nigrofasciata* | KJ561853.1 |
|  |  | *Oreochromis niloticus* | NM_001279683.1 |
| Osteoglossiformes | Mormyroidea | *Gnathonemus petersii* | KJ561851.1 |
|  | Mormyroidea | *Campylomormyrus compressirostris* | KF860194.1 |
|  | Mormyroidea | *Petrocephalus soudanensis* | TR451798 |
|  | Notopteridae | *Chitala chitala* | AIG92916.1 |
|  | Osteoglossidae | *Scleropages jardinii* | KJ561850.1 |
|  | Osteoglossidae | *Scleropages formosus* | KKX00340.1 |
|  | Pantodontidae | *Pantodon buchholzi* | AIG92915.1 |
| Perciformes | Scombridae | *Thunnus albacares* | AAG02112.1 |
| Salmoniformes | Salmonidae | *Salmo salar* | ACM09229.1 |
| Salmoniformes | Salmonidae | *Oncorhynchus mykiss* | BAI45225.1 |
| Tetraodontiformes | Tetradontoidea | *Takifugu rubripes* | XM_003976046.2 |
|  |  | *Tetraodon nigroviridis* | AJ628044.1 |

Table S2 Statistics for amplified genes for each species. Note: ✓ = successfully amplified, ✗ = no amplification despite numerous attempts or fail to find in transcriptome, - = did not try to amplification in our samples. ★ represents sequences downloaded from transcriptome or genome.

| Species | Samples type | *Mb* | *Hba* | *Hbb* | *Ngb* |
| --- | --- | --- | --- | --- | --- |
| *Brachyhypopomus* *gauderio* skeletal muscle | RNA | ✓ | ✓ | ✓ | - |
| *Brachyhypopomus* *gauderio* brain | RNA | - | - | - | ✓ |
| *Gymnorhamphichthys sp.* skeletal muscle | Genomic DNA | ✓ | ✗ | ✗ | ✓ |
| *Gymnotus cylindricus* skeletal muscle | RNA | ✗ | ✓ | ✓ | - |
| *Gymnotus omarorum* skeletal muscle | RNA | ✗ | ✓ | ✓ | - |
| *Gymnotus omarorum* EO | RNA | ✗ | ✗ | ✗ | - |
| *Gymnotus omarorum* heart | RNA | ✗ | ✗ | ✗ | - |
| *Microsternarchus bilineatus* skeletal muscle | Genomic DNA | ✓ | ✓ | ✓ | ✓ |
| *Rhamphicthyes marmoratus* skeletal muscle | RNA | ✓ | ✗ | ✓ | - |
| *Steatogenys elegans* skeletal muscle | RNA | ✓ | ✓ | ✓ | - |
| *Eigenmannia virescens* brain | RNA | - | - | - | ✓ |
| *Eigenmannia virescens* | EO/muscle transcriptome | ★ | ★ | ★ | - |
| *Electrophorus electricus* | Genome | ★ | ★ | ★ | ★ |
| *Apteronotus albifrons* | Skeletal muscle/spinal cord transcriptome | ★ | ★ | ★ | ★ |
| *Parapteronotus hasemani* | Skeletal muscle/spinal cord transcriptome | ★ | *-* | *-* | *-* |
| *Sternopygus macrurus* | EO/muscle transcriptome | ★ | ★ | *-* | ★ |

Table S3: List of primers used to amplify the coding regions of globin genes in this study.

| Gene | Nest PCR 1 | Nest PCR 2 | Melting Temp (°C) | Product Size |
| --- | --- | --- | --- | --- |
| *Hba* | F: CCAGAGTSTSAGTYGARAAG  R: TCAGACRTRDTYATGTCAGS | F: ATGAGTCTCWCTSCKAAGGA  R: RCGRTACTTCTCRGACAGAG | 50-56 | 1200bp |
|  | F: ATGAGTCTCWCTSCKAAGGA  R: RCGRTACTTCTCRGACAGAG | F: TRTSRTBAAGKCYTSCTGGG  R: VGYTCTGGAAGAACTTGTCW | 50-56 | 1200bp |
|  | F: ATGAGTCTCWCTSCKAAGGA  R: RCGRTACTTCTCRGACAGAG | F: GGSYAARRYBDSCYCMAAGG  R: AACTTGTCDACWGMSAHGTG | 50-56 | 450bp |
| *Hbb* | F: ATGGTTGAGTKGACAGAMGC  R: TGATACTGYTTGCYGAGAGC | F: GCGYRGYGCTATCGYYARCC  R: AGACACRACWWCRGCCAWGA | 50-56 | 1200bp |
|  | F: CGTCGCTATGGTTGAGTKGA  R: TGKTCTTAMATGGAGCYGTT | F: CTATCGYYARCSTGTGGGGW  R: AGCAGASRCRACWDCRGMCA | 50-56 | 450bp |
| *Mb* | F: ATGKCTGACTWYGAYMTSATYCTSR  R: ACCRGCGWAGCCSARCTCCTTGTAR | F: YWCAMBGRHTACGGAGGAGMRRTKC  R: WADCYRTCRRYSTCRBYGRTGACVV | 50-56 | 1500bp |
|  | F: ATGKCTGAYYWYGAYMTSRT  R: ACCRGCRWAKCCVADCTCCT | F: YCTSRMNDKCTGGSSVVMBR  R: TGTARHADCYRTCRRYSTCV | 50-56 | 400bp |
| *Ngb* | F: ATGGAGGAACTCTCMGGCAA  R: TCAGTTGGAYTTGTGCTCYC | F: AGACAAACAACTGATCCGGG  R: CGTTCTTGGCMYAGCCTCTG | 50-56 | 500bp |
|  | F: AAAGAYAARSRWCTSATCCG  R: TCAGTTGGAYTTGTGYTCHCC | F: CTGGGAGARHCTGGGVAAGA  R: TTYTTGGCMYARCCTCKRGT | 50-56 | 500bp |
|  | F: AAAGAYAARSRWCTSATCCG  R: TCAGTTGGAYTTGTGYTCHCC | F: VAAGAACAAGGTBCCRCAYG  R: GTGYTCHCCRTTYTTGGCMY | 50-56 | 500bp |

Table S4 Positive Selection Detected by Site Models in PAML

| Gene | Model | Ln | 2ΔLn | *p* | Parameter | Positive selected sites |
| --- | --- | --- | --- | --- | --- | --- |
| *Hbb* | M8 | 1899.524 |  |  | ω =3.230 | 133-0.996* |
|  | M8a | 1903.186 | 7.324 | 0.006 | ω =1 |  |

Table S5 Positive Selection Detected by Branch Models in PAML

| Gene | Model | Ln | 2Ln | *p* | Parameter estimates |
| --- | --- | --- | --- | --- | --- |
| *Hba* | M1 | 3042.036 |  |  | ω variation for each branch |
|  | M0 | 3089.857 | 95.642 | <0.001 | ω = 0.217 |
| *Hbb* | M1 | 3210.926 |  |  | ω variation for each branch |
|  | M0 | 3247.822 | 73.792 | <0.001 | ω = 0.296 |
| *Mb* | M1 | 5246.555 |  |  | ω variation for each branch |
|  | M0 | 5322.174 | 151.238 | <0.001 | ω = 0.218 |
| *Ngb* | M1 | 2219.199 |  |  | ω variation for each branch |
|  | M0 | 2230.599 | 22.8 | 0.35 | ω = 0.075 |

Table S6 Raw and Processed Reads from Each Tissue

| Organ | # of raw PE reads | # of processed PE reads | % of retained reads |
| --- | --- | --- | --- |
| Brain | 45016401 | 40415858 | 89.8 |
| Electric Organ | 64399957 | 54886344 | 85.2 |
| Heart | 48975257 | 43750876 | 89.3 |
| Muscle | 42810723 | 37550957 | 87.7 |
| Total | 201202338 | 176604035 | 87.8 |

Table S7 Evaluation of transcriptome completeness against the Vertebrates, Metazoans and Eukaryotes datasets.

|  | Dataset | | | | | |
| --- | --- | --- | --- | --- | --- | --- |
|  | Vertebrata | % | Metazoa | % | Eukaryotes | % |
| Complete Single-copy BUSCOs | 529 | 17.5 | 134 | 15.9 | 66 | 15.4 |
| Complete Duplicated BUSCOs | 1650 | 54.6 | 585 | 69.4 | 291 | 67.8 |
| Fragmented BUSCOs | 379 | 12.5 | 109 | 12.9 | 50 | 11.7 |
| Missing BUSCOs | 465 | 15.4 | 15 | 1.8 | 22 | 5.1 |
| Total BUSCO groups searched | 3023 |  | 843 |  | 429 |  |
