# Supplementary material for: Molecular evolution of globin genes in Gymnotiform electric fishes: relation to hypoxia tolerance
Source: BMC Evol Biol. 2017 Feb 13;17:51. doi: 10.1186/s12862-017-0893-3 (PMC5307702; doi:10.1186/s12862-017-0893-3)
Supplement: Additional file 2: Figure S1. — Sequences alignment of globin genes. Figure S2 The phylogenetic trees constructed from nucleotide sequences used maximum likelihood tests. (ZIP 729 kb) [file 12862_2017_893_MOESM2_ESM.zip › Fig. S1.pdf]

Figure S1. Alignment of globin genes

Hba

```
                               1
Sternopygus_macrurus           ATGAGTCTCT CTCCTAAGGA CAAAGCTGTG ATGAAGTCCC TGTGGGGCAA
steato_elegans                 ---------- ---------- ---------- ------TCTC TCTGGAGCAA
Microsternarchus_bilineatus    ---------- -----AAGGA CAAGGCTGTG ATTAAGTCCC TCTGGGGCAA
Brachyhypopomus_gauderio       ---------- -----AAGGA CAAGTCTGTG GTTAAGGCCT TCTGGGGCAA
Gymnotus_omarorum              ---------- -----AAGGA CAAATGCGTG GTTAAGTCTC TCTGGGCTAA
Gymnotus_cylindricus           ---------- -----AAGGA CAAATGTGTG GTTAAGTCTC TCTGGGCTAA
Electrophorus_electricus       ATGAGTCTCA CTGCTAAGGA CAAGTCTATC GTCAAGGCCT TCTGGGGCAA
Eigenmannia_virescens          ATGAGTCTCT CTGCGAAGGA CAAGTCTGTG ATTAAGGCCC TGTGGGGCAA
Apteronotus_albifrons          ATGAGTCTCT CTCCTAAGGA CAAATCTGTG ATGAAGTCCC TGTGGGGCAA

                              51
Sternopygus_macrurus           AATCGCCCCC AAGGCCGATG AAATCGGAGC TGAAGCTTTC GGCAGAATGC
steato_elegans                 AGTTTCCTCC AAGGCCGATG AAATTGGGGC TGAAGCTTTT GGCAGAATGC
Microsternarchus_bilineatus    AATTGCCCCC AAGGCCGATG AAATCGGAGC TGAAGCTTTT GGCAGAATGC
Brachyhypopomus_gauderio       AATTGCCTCC AAGGCCGATG AAATCGGAGC TGAAGCTTTT GGCAGAATGC
Gymnotus_omarorum              AATTTCCCCC AAGGCCGATG AAATAGGGGC GGAAGCTCTT GGCAGAATGC
Gymnotus_cylindricus           AATTTCCCCC AAGGCCGATG AAATCGGGGC GGAAGCTCTT GGCAGAATGC
Electrophorus_electricus       AGCGTCCTCC AAGGCCGATG ACATCGGGGC TGAAGCTTTT GGCAGAATGC
Eigenmannia_virescens          AATTGCCCCC AAGGCCGATG AAATTGGGGC TGAAGCTTTG GGCAGAATGC
Apteronotus_albifrons          AATTGCCCCA AAGGCCGATG AAATCGGGGC TGAAGCTTTG GGCAGAATGC

                             101
Sternopygus_macrurus           TCACCGTATC CCCCCAGACA AAGACCTACT TCTCCCATTG GTCCGACCTG
steato_elegans                 TCCCCGTATA CCCCCAAACA AAGACCTATT TCTCCCACTG GTCCGACCTG
Microsternarchus_bilineatus    TCACCGTCAG CCCCCAGACT AAGACCTACT TCTCCCACTG GTCCGACCTG
Brachyhypopomus_gauderio       TCATCGTCAG CCCCCAGACT AAGACCTACT TCTCTCACTG GTCCGACCTG
Gymnotus_omarorum              TCGCCGTAAA CCTCCAGACA AGATCCTACT TCACCCATTT GTCCGACCTG
Gymnotus_cylindricus           TCGCCGTAAA CCTCCAGACA AGATCCTACT TCTCCCATTT GTCCGACCTG
Electrophorus_electricus       TCACCGTATA CCCCCAGACA AAGACTTACT TCGCCAGTTG GTCCGACCTG
Eigenmannia_virescens          TCGTCGTATA CCCCCAGACA AAAACCTACT TCTCCCATTG GTCCGACCTG
Apteronotus_albifrons          TCACCGTTTA CCCCCAGACA AAGACCTACT TTTCCCACTG GTCAGACTTG

                             151
Sternopygus_macrurus           AGCCCCGGAT CCGCTCCCGT GAAGAAGCAC GGTAAAACCG TCATGGGCGG
steato_elegans                 ACCCCCGGAT CCCCTTCTGT GAAGAAGCAC GGAAAGGTCG TCATGGGCGG
Microsternarchus_bilineatus    AGCCCCGGGG CCGCTCCCGT GAAGAAGCAC GGAAAGACTG TGATGGGCGG
Brachyhypopomus_gauderio       CACCCCGGAT CCGGTCCCGT CAAGAAGCAC GGAAAGGTTG TGATGGGCGG
Gymnotus_omarorum              AGCCCCGGAT CAGCTCAAGT GAAGAATCAC GGAAAAGTCG TCATGGGCGG
Gymnotus_cylindricus           AGCCCCGGAT CAGCTCAAGT GAAGAATCAC GGAAAAGTCG TCATGGGCGG
Electrophorus_electricus       AGCCCCGGAT CCGCTGCTGT GAAGAAGCAC GGAAGAACTA TCATGGGCGG
Eigenmannia_virescens          AGCCCCGGGT CCGCCCCTGT GAAGAAGCAC GGGAAAACTG TCATGGGCGG
Apteronotus_albifrons          AGCCCCGGAT CCGCCCCTGT GAAGAAGCAC GGAAAGGTCA TCATGGGTGG

                             201
Sternopygus_macrurus           AGTCGCTGAG GCGATTTCCA AAATTGATGA CCTTGCTGGC GGTTTGGCCT
steato_elegans                 AGTCGCTGAG GCGGTTTCCA AAATCGATGA CCTTGCTGGA GGGTTGGCCT
Microsternarchus_bilineatus    AGTCGCTGAG GCGGTTTCCA AGATCGATGA CCTGTGTGGG GGTCTGGCCT
Brachyhypopomus_gauderio       AGTCGCTGAG GCGGTTTCCA AAATTGATGA CCTTACCGGT GGGCTGGCCG
Gymnotus_omarorum              AGTCGCTGAG GCGGTTGCCA AAATCGATGA CCTTGCTTGC GGTTTGTCCG
Gymnotus_cylindricus           AGTCGCTGAG GCGGTTGCCA AAATCGATGA CCTTGCTTGC GGTTTGTCCG
Electrophorus_electricus       AATCGCTGAG GCGGTTGGCC ACATTGATGA CCTTACTGGC GGTTTGGCCT
Eigenmannia_virescens          AGTCGCTGAA GCGATTGCCA AGATTGATGA TCTTGCTGGC GGTTTGGCCT
Apteronotus_albifrons          AATCGCTGAA GCGATTTCCA AAATTGATGA CCTTTGTGGC GGATTGAGCG

                             251
Sternopygus_macrurus           CGCTCAGCGA ACTGCACGCG TTCAAGCTGC GGGTGGACCC TGCTAACTTC
steato_elegans                 CTCTCAGCGA ACTGCACGCG TTCCAGCTGC GGGTGGACCC TGCTAACTTC
Microsternarchus_bilineatus    CGCTCAGCGA GCTGCACGCC TCCAAGCTGC GCGTGGACCC TGCCAACTTC
Brachyhypopomus_gauderio       CGCTCAGCGA ACTGCACGCG TCCAAGCTGC GGGTGGACCC TGCTAACTTC
Gymnotus_omarorum              CGCTCAGCGA GCTGCACGCA TCCAAGCTGC AGGTGGACCC CGCTAACTTC
Gymnotus_cylindricus           CGCTCAGCGA GCTGCACGCA TCCAAGCTGC AGGTGGACCC CGCATACTTC
Electrophorus_electricus       CGCTCAGTGA ACTGCACGCT TTCAAGCTGC GGGTGGACCC TGCTAACTTC
Eigenmannia_virescens          CGCTCAGTGA ACTGCACGCG TTCAAGCTGC GGGTAGATCC TGCTAACTTC
Apteronotus_albifrons          CGCTCAGCGA ACTGCACGCA TTCAAGCTGC GGGTGGATCC TGCAAACTTC

                             301
Sternopygus_macrurus           AAGATTCTGG CGCACAACAT CATACTGGTG TTGGCCATGT ACTTCCCCGC
steato_elegans                 AAGATTCTTG CGCACTGCCT CATCGTGGTG TTGGCCATGT ATTTCCCTGC
Microsternarchus_bilineatus    AAGGTTCTGG CGCACAACAT CATCCTGGTG CTGGCCATGT ACTTCCCTGC
Brachyhypopomus_gauderio       AAGATTCTTG CGCACAACAT CATACTGGTG CTGGCCATGT ACTTCCCTGC
Gymnotus_omarorum              AAGGTTCTTG CGTACAACAT CATACTGGCG ATTGCCATGT ACTTCCCTGC
Gymnotus_cylindricus           AAGATTCTTG CGCTCAACCT CATACTGGCG ATTGCCATGT ACTTCCCTGC
Electrophorus_electricus       AAGATTCTTG CGCACAACCT CATAGTGGTC TTGGCCTTGT TCTTCCCTGC
Eigenmannia_virescens          AAGATTTTGG CGCACAACAT CATACTGGTC TTGGGCATGT ACTTCCCCGG
Apteronotus_albifrons          AAGATCCTGG CGCATAGCGT TATTGTGGTC TTGGCCATGT ATTTCCCTGC
```

```
                              351
Sternopygus_macrurus          GGACTTCACT CCTGAGGTTC ACGTGTCTGT AGACAAGTTC TTCCAGAACT
steato_elegans                AGATTTCACT CCTGAGGTGC ACGTGTCTGT AGATAAGTTC TTTCAGAACT
Microsternarchus_bilineatus   GGATTTCACT CCTGAGGTGC ACGTCTCTGT AGAC------ ----------
Brachyhypopomus_gauderio      GGACTTCACT CCTGAGGTGC ACTTGTCTGT AGACAAGTTC TTCCAGAATC
Gymnotus_omarorum             TGATTTCACA CCTGAGGTTC ACTTGTCTGT AGACAAGTTC TTCCACAACG
Gymnotus_cylindricus          GGATTTCACA CCTGAGGTTC ACGTGTCTGT AGACAAGTTC TTCCAGAACG
Electrophorus_electricus      GGATTTCACT CCTGAGGTTC ACATGGCTGT TGACAAGTTC TTCCAGAACG
Eigenmannia_virescens         AGATTTCACT CCTGAGGTTC ACGTGTCTGT AGACAAGTTC TTCCAGAACC
Apteronotus_albifrons         CGACTTCACT CCAGAGGTTC ACGTCTCTGT AGACAAGTTC TTCCAGAGCC

                              401
Sternopygus_macrurus          TGGCCTGGGC TCTGTCTGAG AAGTACCGC
steato_elegans                TGGCCTGGGC TCTGT----- ---------
Microsternarchus_bilineatus   ---------- ---------- ---------
Brachyhypopomus_gauderio      TGGCCTGGGC TCTGT----- ---------
Gymnotus_omarorum             TGGCCTGGGC TCTGT----- ---------
Gymnotus_cylindricus          TGGCCTGGGC TCTGTC---- ---------
Electrophorus_electricus      TGGCCTGGGC TCTGTCCGAG AAGTACCGC
Eigenmannia_virescens         TGGCCTGGGC TCTGTCTGAG AAGTATCGC
Apteronotus_albifrons         TGGCCTGGGC TCTGTCCGAG AAGTACCGT
```

Hbb

```
                             1
Steatogenys_elegans          ATGGTTGAGT GGACAGACGC CGAGCGCAGC GCTATCGCTA GCCTGTGGGG
Brachyhypopomus_gauderio     ---------- --------GC CGAGCGCAGC GCTATCGTCA GCGTGTGGGG
Gymnotus_cylindricus         ---------- ---------- ---------- ---------- ---TGTGGGG
Gymnotus_omarorum            ATGGTTGAGT GGACAGATGT CGAGCGCAGT GCTGTCGCCA GCCTGTGGGG
Rhamphicthyes_marmoratus     ---------- ---------- ---------C ACTATCGTTA GCCTGTGGGG
Microsternarchus_bilineatus  ------GAGT GGACCGAAGC CGAGCGCAGC GCCATCGTCA GCATCTGGGG
Eigenmannia_virescens        ATGGTTGAGT GGACAGACGC CGAGCGCAGT GCTATCGCCA GCCTGTGGGG
Electrophorus_electricus     ATGGTTGAGT TGACAGAAGC CCAGCGTGGT GCTATCGTTA ACCTGTGGGG
Apteronotus_albifrons        ATGGTTGAGT GGACAGACGC CGAACGCAGT GCTATCGTTA GCGTGTGGGG

                             51
Steatogenys_elegans          ACAACTCAGT GTAGATGAAA TCGGACCTCA GGCTCTGGCC AGGGTTCTGG
Brachyhypopomus_gauderio     TCAGCTCCAC GCAGATGAAG TCGGAGCTCA TGCTATTGCA AGGCTCCTGA
Gymnotus_cylindricus         ACAGCTCAGT GTAGACGAAA TCGGACCTCA GACTTTAGCC AGGTTCCTGA
Gymnotus_omarorum            ACAGCTCAGT GTAGACGAAA TCGGACCTCA GGCTTTAGCC AGGCTCCTGA
Rhamphicthyes_marmoratus     ACACCTCAGT GTAGAGGAAA CCGGACCTCA GGCTTTGGCC AGGCTTCTGA
Microsternarchus_bilineatus  TCAGCTCAGT GTTGAGGAAA TCGGACCTCA GGCTTTGGCC AGGCTTATGA
Eigenmannia_virescens        ACAGCTCAGT GTAGATGAAA TCGGACCACA GGCTCTGGCC AGGCTTCTGG
Electrophorus_electricus     ACATCTGAGT CCAGATGAAA TCGGACCTCA GGCTTTGGCC AGGCTTCTGA
Apteronotus_albifrons        ACAGATCAGT ATCGATGAAA TCGGACCCCA GGCTTTGGCC AGGCTTCTGA

                             101
Steatogenys_elegans          TCGTATTCCC ATGGACACAG AGGTACTTCG CTGCCTTTGG TAACATTTCC
Brachyhypopomus_gauderio     TTGTATACCC ATGGACACAG AGATACTTCT CCGCCTTTGG AAACATATCC
Gymnotus_cylindricus         TAGTATACCC ATGGACACGG AGATACTTCG CTTCCTTTGG TGACATCTCC
Gymnotus_omarorum            TTGTATACCC ATGGATACAG AGATACTTCC CTTCCTTTGG TGACCTCTCC
Rhamphicthyes_marmoratus     TTGTATACCC ATGGACACAG AGATACTTCG CTTCCTTTGG TAACCTATCC
Microsternarchus_bilineatus  TTGTATACCC ATGGACACAG AGATACTTCG CGTCCTTTGG TAACATATCC
Eigenmannia_virescens        TCGTATACCC ATGGACACAG AGATACTTCG CTACCTTTGG TAACCTGTCC
Electrophorus_electricus     TTGTATACCC ATGGACACAG AGATACTTCG CTTCCTTTGG TAACATATCC
Apteronotus_albifrons        TTGTATTCCC ATGGACACAG AGATACTTCG CGTCCTTTGG TAGCCTGTCT

                             151
Steatogenys_elegans          AGCCCCGCCG CCATCATGGG GAACCCCAAA GTGGCGGCGC ACGGCAAAGT
Brachyhypopomus_gauderio     AGCCCCGCTG CCATTATGGG GAACCCCAAA GTTGCGGCGC ACGGTAAAGT
Gymnotus_cylindricus         ACCCCCGCTG CCGTTATGGG GAACCCCAAA GTAGCGGCGC ACGGGAAAAT
Gymnotus_omarorum            ACCCCCGCTG CCATTATGGG GAACCCCAAA GTAGCGGCGC ACGGGAAAAT
Rhamphicthyes_marmoratus     AGCCCCGCTG CCATTTTGGG GAACCCCAAA GTGGCGGCTC ACGGTAAAGT
Microsternarchus_bilineatus  AGCCCCGCTG CCATTATGGG GAACCCCAAA GTTGCGGCGC ACGGTAAAGT
Eigenmannia_virescens        AGCCCCGCTG CCATTCAAGG CAATCCTAAA GTGGCGGCAC ACGGTAAAAC
Electrophorus_electricus     AGCGCCGCGG CCATTATGGG GAACCCCAAA GTGGCGGCGC ACGGTAAAGT
Apteronotus_albifrons        AGCCCCGCTG CCATTATGGG TAACGCCAAA GTGGCGGCGC ACGGAAAAAT

                             201
Steatogenys_elegans          CGTCATGGGT GCTCTGGAAA AAGGCGTGAA GAACCTGGGC GGCATCAAGA
Brachyhypopomus_gauderio     TGTCATGGGA GCTCTGGAAA AAGGCGTGAA GAATCTGAAC AACCTCAAGG
Gymnotus_cylindricus         CGTCATGGGT GCTCTGGAAA ACGGCGTGAA GAACCTGGGC AACATCAAAA
Gymnotus_omarorum            CGTCATGGGT GCTCTGGAAA ACGGCGTGAA GAACCTGGGC AACACCAAAA
Rhamphicthyes_marmoratus     CGTCATGGGT GCGCTGGACA AAGGCGTGAA GAACCTGAGC GGCATCAAGG
Microsternarchus_bilineatus  CGTCATGGGT GCTCTGGACA AAGGTGTGAA GAACCTGAAC AACATTAAGG
Eigenmannia_virescens        CGTCATGGGA GCGCTGGAGA AGGGCGTGAA GAATCTGGGC GCCATCAAGG
Electrophorus_electricus     CGTCGTGGGT GCTCTGGACA AAGGCGTGAA GAACCTGAAC AACATCAAGG
Apteronotus_albifrons        CGTCATGGGT GCTCTGGATA AAGGTGTAAA GCACCTGGAC AGCATCAAGG

                             251
Steatogenys_elegans          GCACCTACTC CGCGCTGAGC GCGATGCACT CTGATAAACT CCACGTGGAT
Brachyhypopomus_gauderio     GCACCTACGC AGCGCTGAGC ACGATGCACT CTGAGAAGCT CCACGTGGAT
Gymnotus_cylindricus         GCAACTACGC TGCGCTGAGC GCGATGCACT CGGACAAACT CCACGTGGAC
Gymnotus_omarorum            GCACCTACGC TGCGCTGAGC GCGATGCACT CGGACAAACT CCACGTGGAC
Rhamphicthyes_marmoratus     GCACCTACTC AGCACTGAGC GCGATGCACT CTGATAAACT CCACGTGGAT
Microsternarchus_bilineatus  GCACCTACGC CGGGCTGAGC GCGATGCACT CCGACAAGCT CCACGTGGAT
Eigenmannia_virescens        CCACCTACTC CGCGCTGAGC ACCATGCACT CTGAAAAACT CCACGTGGAT
Electrophorus_electricus     GTACCTACGC TGCGCTGAGC ACTATCCACT CTGAAAAACT CCACGTGGAT
Apteronotus_albifrons        CCACCTACTC CGCGCTGAGC AAAATGCACT CTGACAAACT CCACGTGGAT

                             301
Steatogenys_elegans          CCCGACAACT TCAGGCTTAT TGCCGAGTGC ATCACCGTAT GTGTGGGCAT
Brachyhypopomus_gauderio     CCCGACAACT TCAGGCTTCT TGCCCACTGC ATCAGCACAA CTATTGCCAT
Gymnotus_cylindricus         CCCGAAAACT TCAGGCTTCT TGCCGACTCC TTGTCCGTGT GTGTGGCCAT
Gymnotus_omarorum            CCCGACAACT TCAGGCTTCT TGCCGAGTCC TTGTCCGTGT GTGTGGCCAT
Rhamphicthyes_marmoratus     CCCGACAACT TCAAGCATAT TGCTGACTGC GTCATCGTAT GTGCCGCCAT
Microsternarchus_bilineatus  CCCGACAACT TCAGGCTTCT TGCCGAGTGC CTCACCGTAT GTGTCGCCAT
Eigenmannia_virescens        CCCGACAACT TCAGGCTTCT TGCCGAGTGC GTGACCGTAT GCGTAGGCAT
Electrophorus_electricus     CCCGACAACT TCAGGCTTCT TGCCGAGTGC TTCACCGTAT GTGTGGCCAT
Apteronotus_albifrons        CCCGACAACT TCAAGCATCT GGCCGAGTGC ATCACCGTAT GTGTGGCCAT
```

```
                               351
Steatogenys_elegans            GAAGCTGGGA CCCTCCGTCT TCAACGCTGA CGCGCAGGAG GCTTGGTGCA
Brachyhypopomus_gauderio       GAAGCTGGGA CACTCCGCCT TCACCCCTGA AGTGCAGGAG GCCATTGAGA
Gymnotus_cylindricus           GAAGCTGGGG CCTTCCGTCT TCACGGCTAA CGCTCAGGAG GCTTGGTCCA
Gymnotus_omarorum              GAAGCTGGGG CCTTCCGTCT TCACGGTTAA CGCTCAGGAG GCTTGGTCCA
Rhamphicthyes_marmoratus       GAAGCTGGGA ---TCCGGCT TCACCCCTGA CGTGCAGGAG GCCTTGACCA
Microsternarchus_bilineatus    GAAGCTGGGA CACTCCGTCT TCAACGCGGA CGCGCAGGAG GCGTGGTGCA
Eigenmannia_virescens          GAAGCTGGGA CCGTCCGTCT TCAACGCTGA CGCGCAGGAA GCTTGGTGCA
Electrophorus_electricus       GAAGCTGGGA CCCTCCGGCT TCAACGCTGA AACGCAGCAC GCTTTGGCCA
Apteronotus_albifrons          GAAGTTCGGA CCCTCGGCCT TCACCCCTGA CGTGCAGGAG GCTTGGCAGA

                               401
Steatogenys_elegans            AGTTCATGGC CGTTGTCGTG TCTGCTCTCA GCAAACAGTA TCAT
Brachyhypopomus_gauderio       AGTTCTTGTC TGCTGTGGCC TCTGCTC--- ---------- ----
Gymnotus_cylindricus           AATTCTTGGC CGCTGTC--- ---------- ---------- ----
Gymnotus_omarorum              AATTCTTAGC GGTGGTCGTG TCCGCTCTCT CCAAA----- ----
Rhamphicthyes_marmoratus       AGTTCCTGGC CG-------- ---------- ---------- ----
Microsternarchus_bilineatus    AGTTCCTGGC CG-------- ---------- ---------- ----
Eigenmannia_virescens          AGTTCTTGGC TGTTGTTGTG TCTGCTCTCA GCAAGCAGTA TCAT
Electrophorus_electricus       AGTTCTTGGC TGAAGTTGTG TCTGCTCTCG GCAAACAGTA TCAT
Apteronotus_albifrons          AGTTCTTGGC TGTTGTTATC TCTGCGCTTT CCAGACAGTA CCAT
```

Mb

```
                              1
Sternopygus_macrurus          ATGGCTGACT TTGACCTCAT CCTCAACTGC TGGCCAACCA TCGAGGCTGA
Apteronotus_albifrons         ATGGCTGACT TCGACCTGGT CCTGAAAAGC TGGCCGGCTG TGGAGGCCGA
Eigenmannia_virescens         ATGGCTGACT TCGACCTGAT CCTGAAGTGC TGGCCCGCTG TGGAGGCTGA
Gymnorhamphichthys_sp.        ---------- ---------- ---------- ---------- ----------
Microsternarchus_bilineatus   ---------- ---------- ---------- ---------- ----------
Brachyhypopomus_gauderio      ATGTCTGACT TTGATATGAT CCTGGCTGTC TGGCCAAAGG TGGAAGCCAA
Rhamphicthyes_marmoratus      ---------- ---------- -----AGGTC TGGCCAAAAG TGGAAGCCGA
Steatogenys_elegans           ---------- ---------- -----AAGTC TGGCCTGCTG TGGAAGCCGA
Electrophorus_electricus      ATGTCTGACT ACGACCTGAT TCTGAAGTGC TGGCCACCCG TGGAAGCCGA
Parapteronotus_hasemani       ATGGCTGACT TCGACCTGGT TCTGAAAAGC TGGCCGGCTG TGGAGGCCGA

                              51
Sternopygus_macrurus          CTACAACGGC TACGGAGGAG AGATTCTGAC CCGTCTGTTC TTGGAGCATC
Apteronotus_albifrons         CTACACCGGT TATGGAGGAG AAGTTCTGAC CCGTCTGTTT TTGGAGCATC
Eigenmannia_virescens         CTACAATGGA TACGGAGGAG AGGTTCTGTC CCGCCTCTTC TTGGATCATC
Gymnorhamphichthys_sp.        ---------- ---------- ------TGGT CCGGCTGTTT ATCGAGCATC
Microsternarchus_bilineatus   ---------- ---------- -----CTGGG CCGGCTGTTT TTGGACCATC
Brachyhypopomus_gauderio      CCTCAAGGAT TACGGAGGAG AGGTGCTGTG GGGTCTGTTT TTGGAACATC
Rhamphicthyes_marmoratus      CTTCACCGGT TTTGGAGGAG AGGTTCTGAC CCGTCTGTTT ATAGAGCATC
Steatogenys_elegans           CTACACTGGT TATGGAGGAG AGGTTCTGAC CCGTCTGTTT ATGGAGTATC
Electrophorus_electricus      CTACACTGGT TACGGAGGAG CAGTTCTGGG CCGTCTGTTT GTCGAACATC
Parapteronotus_hasemani       CTACACCGGT TACGGAGGAG AAGTTCTGAC CCGTCTGTTT TTGGAGCATC

                              101
Sternopygus_macrurus          CCGACACCCA GAAGGTCTTC CCCAAGTTCT CTAACATTCC CCGCGGCAGC
Apteronotus_albifrons         CAGAGACACA GAAGGTCTTC CCCAGGTTCG TCAACATCCC CCGCGGTAGC
Eigenmannia_virescens         CTGAGACCCA GAAGGTTTTC CCCCGTTTCG TGAGTATCCC TCGTGGAAGC
Gymnorhamphichthys_sp.        CTGAGACCCA GAAGCTGTTC CCAAAGTTCG TGGGGATCCC ACGCGGCGGC
Microsternarchus_bilineatus   CTGATAGCCA GAAGTTTTTC CCGAGGTTCC AGAGTATCAC ACGCGCTAAC
Brachyhypopomus_gauderio      CTGAAAGCCA GAAATACTTC CCAAAGTTCA GAGATATCCC GCAGGGTGAG
Rhamphicthyes_marmoratus      CTGAGACCCA GCAGCTCTTC CCAAGGTTCG CGGGGATCTC ACAAGGTAAC
Steatogenys_elegans           CTGAGACCCA GTCACTCTTC CCAAAATTCA AGAGTATCCC ACAGAGTAAC
Electrophorus_electricus      CTGACACCAT CAAGTTCTTC CCTAAGTTCG CGAATATCCC ACGCGGTAGC
Parapteronotus_hasemani       CAGAGACACA GAAGGTCTTC CCCAGGTTCG CCAACATCCC TCGCGGTAGC

                              151
Sternopygus_macrurus          CTGGCTGGGA ATGCTGACAT TGCAGCCCAC GGAACCGTCG TGCTGAAGAA
Apteronotus_albifrons         TTGGCTGGTA ATGCTGACAT CGCAGCTCAC GGAGTGACAG TGCTGAAGAA
Eigenmannia_virescens         GTGGTGGGCA ACCCCGACAT CGCCGCCCAC GGGGCCACTG TGCTGAAAAA
Gymnorhamphichthys_sp.        CTGGCGGGCA ACTCCGACAT CGCGGCCCAC GGGTGCACCG TACTGAAGAA
Microsternarchus_bilineatus   ATTCCAGGGA ATGCTGATAT CGCGTCCCAC GGATGCACAG TGCTGAAGAA
Brachyhypopomus_gauderio      CTTCAAGGAA ATGCTGCTAT TGCGGCTCAT GGATGCACAG TGCTGACGAA
Rhamphicthyes_marmoratus      CTAGCAGGCA ACGCCGATGT TGCGGCCCAC GGATGCACTG TGCTGAAGAA
Steatogenys_elegans           CTAGCAGGCA ATCCTGACAT TGCAGCCCAT GGTGTCACTG TACTGAGGAA
Electrophorus_electricus      CTGGCGGGGC ATCCTGACGT AGCGGCCCAC GGAGCCACTG TGCTGAAGAA
Parapteronotus_hasemani       TTGGCTGGTA ATGCTGACAT CGCAGCTCAC GGAGTGACGG TGCTGAAGAA

                              201
Sternopygus_macrurus          GCTCACCGAA CTGGTCAAGG CCAAGGGTAA CCACGCCTCC GTACTCAAGC
Apteronotus_albifrons         GCTGGGGGAG CTTCTGAAGG CCAAGGGGAA CCACGCTGCA GCACTCAAGC
Eigenmannia_virescens         GCTGGGTGAG CTGGTTAAAG CCAAGGGCAA CCACGCTTCC GCCCTCAAAC
Gymnorhamphichthys_sp.        GCTGGGCGAC CTGCTCAAAG CCAAGGGGAA CCACGCCGCG GTCCTCAAGC
Microsternarchus_bilineatus   ACTCGGTGAG GTGATCAAGG CAAAGGGGAA CCACGGCCCC ATCATCAAAG
Brachyhypopomus_gauderio      GCTTGGTGAG CTGGTGAAGG CGAAGGGGAA CCACGCCAGC GTCCTCAAAC
Rhamphicthyes_marmoratus      GCTCGGCGAA CTGCTCAAGG CCAAGGGGAA CCACGGCTCA ATCCTCAAAC
Steatogenys_elegans           ACTCGGTGAG CTTGTAAAGG CCAAAGGCAA CCATGCCCCA ATTCTCAAAC
Electrophorus_electricus      GGTAGCCGAG CTGGTCAAAA CCAAGGGGAA CCACACCGGT ATCCTCAAGA
Parapteronotus_hasemani       GCTGGGGGAG CTCCTGAAGG CCAAGGGGAA CCACGCCGCA GCACTCAAAC

                              251
Sternopygus_macrurus          CGCTGGCCAC CAGACATGCC AACGTACACA AGGTCTCCAT CGCCAACTTC
Apteronotus_albifrons         CCCTCGCCAC CAGCCACGCC AACATACACA AGGTCACCAT CGCCAACTTC
Eigenmannia_virescens         CTCTCGCCAC CAGCCACGCC AACATACACA AGGTCACCAT CGCCAACTTC
Gymnorhamphichthys_sp.        CTCTCGCCAC CACCCACGCC AACACGCACA AAATCGCCCT TGGCAACTTC
Microsternarchus_bilineatus   CTCTCGCCAC TACCCACGCC AACCAGCACA AGATCCCCCT GGCCCAGTTC
Brachyhypopomus_gauderio      CTCTCGCCAC TACCCACGCC AACCAGCACA AAATCCCCAT TAACATGTTC
Rhamphicthyes_marmoratus      CTCTCGCCAC CACTCACGCC AACACGCACA AAATCACTCT TGTCAACTTC
Steatogenys_elegans           CACTCGCGAC CACCCATGCC AACACGCACA AGATCAGCCT TGGCAACTTC
Electrophorus_electricus      CTCTCGCCAC CAGCCACGCC AACCAGCACA AGATCCCCAT TATCAACTTC
Parapteronotus_hasemani       CCCTCGCCAC CAGCCACGCC AACATTCACA AGGTCACCAT CGCCAACTTC
```

```
                              301
Sternopygus_macrurus           AACCTGATCA GTGAGATCCT CGTCAAGGTG CTGACCGAGA AGGCAGGAAT
Apteronotus_albifrons          AAGCTGATCA CCGAAATCCT CATCAAGGTT CTGGCTGAGA AGGCAGGAAT
Eigenmannia_virescens          AAGCTGATCA GTGAGATTCT GGTCAAGGTG CTGGCTGAAA AGGCGGGAAT
Gymnorhamphichthys_sp.         AAGCTGATCA CCGAGGTCCT CATCAAGGTG CTGCAGGAGA AAGCTGGGAT
Microsternarchus_bilineatus    AAGGTGATCA CTGAAGTGCT CATCAAGGTC CTGCACGAGA AGGCTGGAAT
Brachyhypopomus_gauderio       AAGTTGATCA CTGAGGTGCT CATCAGTGTG CTGCAGAAAA AGGCTGGAAT
Rhamphicthyes_marmoratus       AAGCTGATCA CCGAGATCCT CGTCAAGGTG CTGCATGAGA AGGCTGGCAT
Steatogenys_elegans            AAGCTGATCA CTGAGGTCCT TATCAAGGTG CTGAAGGAGA AGGCTGGAAT
Electrophorus_electricus       AAGCTGCTCA GCGAGTGCCT GTGCGTTGTG ATGAAAGAGA AGGCAGGAGC
Parapteronotus_hasemani        AAGCTGATCA CTGAGATCCT CATCAAGGTT CTGGCTGAGA AGGCAGGAAT

                              351
Sternopygus_macrurus           CGACACTGCC ACCCAGGACG CCCTGAGGAG AGTCCTGGCC GCCGTCATCA
Apteronotus_albifrons          CGATGGCGCT ACCCAGGATG CCGTGCGGAG AGTGATGGGC GTGGTCATCA
Eigenmannia_virescens          CGACGGAGCC ACCCAGGACG CCCTGAGGAG AGTCCTGGGC ATCGTCATCA
Gymnorhamphichthys_sp.         CGACGGAGCT ACCCAGGATG CCCTGCGGAG AGTGATGGGC GTGGTCATCA
Microsternarchus_bilineatus    CGATGGAGCT ACCCAGGATG CTCTGAGGAG AGTGATGGGC GTGGTCATCA
Brachyhypopomus_gauderio       CGACAAAGCT ACCGCGGAAG CTTTTAGGAG AGTGATGACC GCGGTCACCG
Rhamphicthyes_marmoratus       TGACGGGGCT ACCCAGGATG CCCTGAGGAG AGTGATGGCG GCGGTCATCA
Steatogenys_elegans            TGACGCTGCT ACCCAGGATG CTCTGAGGAA AGTTATGGGC GTGGTCATCA
Electrophorus_electricus       TGATGCTGCT ACCCAGGATG CCCTCAGGAG AGTGCTCAGC TGTGTCACCA
Parapteronotus_hasemani        CGATGGTGCT ACCCAGGATG CCGTGCGGAG AGTGATGGGC GTGGTCATCA

                              401
Sternopygus_macrurus           GTGACACTGA CGGCTATTAC AAGGAGCTCG GCTTCGCTGG T
Apteronotus_albifrons          ACGACATCGA CGGATATTAC AAGGAGCTTG GCTTCGC--- -
Eigenmannia_virescens          ACGACATCGA CGGATATTAC AAGGAGCTCG GCTTCGCTGG T
Gymnorhamphichthys_sp.         ACGACATCGA CGGATA---- ---------- ---------- -
Microsternarchus_bilineatus    ATGACATCGA CGGATG---- ---------- ---------- -
Brachyhypopomus_gauderio       CTGACATCGA CAGTTACTAC AAGGAGCTGG GCTTCGCCGG T
Rhamphicthyes_marmoratus       ACGACATCGA CGGATA---- ---------- ---------- -
Steatogenys_elegans            ACGACATTGA TGGATA---- ---------- ---------- -
Electrophorus_electricus       GCGAGGTTGA TGGATTTTAC AAGGAGTTGG GCTACGCTGG T
Parapteronotus_hasemani        ACGACATCGA CGGATATTAC AAGGAGCTTG GCTTCGCCGG T
```

Ngb

```
                                 1
Electrophorus_electricus         ATGGAGGAAC TCTCAGGCAA AGACAAACAA CTGATCCGGG ACAGCTGGGA
Sternopygus_macrurus             ATGGAGGAAC TCTCCGGCAA AGACAAACAA CTGATCCGGG ACAGCTGGGA
Apteronotus_albifrons            ATGGAGGAAC TCTCAAGCAA AGACAAACAA CTGATCCGGG ACAGCTGGGA
Eigenmannia_virescens            ---------- -----GGCAA AGACAAACAA CTGATCCGGG ACAGCTGGGA
Gymnorhamphichthys_sp.           ---------- ---------- ---------- ---------- ----------
Microsternarchus_bilineatus      ---------- ---------- ---------- ---------- ----------
Brachyhypopomus_gauderio         ---------- ---------- ---------- ---------- ----------

                                51
Electrophorus_electricus         GAAACTGGGA AAGAACAAGG TCCCACACGG CATTGTCATG TTTACCAGGC
Sternopygus_macrurus             GAGCCTGGGC AAGAACAAGG TCCCGCACGG CATTGTCATG TTTACCAGGT
Apteronotus_albifrons            GAGCCTGGGC AAGAACAAGG TCCCACATGG CATTGTCATG TTTACCAGGC
Eigenmannia_virescens            GAGCCTGGGC AAGAACAAGG TTCCACATGG CATTGTCATG TTTACCAGGT
Gymnorhamphichthys_sp.           ---------- AAGAACAAGG TCCCACACGG CATTGTCATG TTTACCAGGC
Microsternarchus_bilineatus      ---------- AAGAATAAGG TTCCACACGG CATTGTCATG TTTACCAGGC
Brachyhypopomus_gauderio         ---------- AAGAATAAGG TTCCACACGG CATTGTCATG TTTACCAGGC

                               101
Electrophorus_electricus         TGTTCGAACT GGACCCGGCT TTGCTCAGCC TCTTCAGCTA CAAGACCAAG
Sternopygus_macrurus             TGTTTGAACT GGACCCAGCT CTGCTCAGCC TCTTCAGCTA CAAAACCAAG
Apteronotus_albifrons            TGTTTGAATT GGACCCAGCT CTGCTCAGCC TCTTCAGCTA CAAAACCAAG
Eigenmannia_virescens            TGTTTGAACT GGACCCGGCT CTGCTCAGCC TCTTCAGCTA CAAAACCAAG
Gymnorhamphichthys_sp.           TGTTTGAACT GGACCCAGCT CTGCTTAGCC TCTTCAGCTA CAAAACCAAG
Microsternarchus_bilineatus      TTTTTGAACT GGATCCAGCT CTACTCAGCC TGTTCAGCTA CAAAACGGAG
Brachyhypopomus_gauderio         TGTTTGAACT GGACCCGGCT CTGCTCAGCC TCTTCAGCTA TAAAACCGAG

                               151
Electrophorus_electricus         TGTGGTGCGG TGCCGGACTG CCTCTCCAGC CCTGAGTTCC TCGACCATGT
Sternopygus_macrurus             TGCGGGGTAG TGCCAGACTG CCTCTCCAGC CCTGAGTTCC TCGAGCATGT
Apteronotus_albifrons            TGCGGCATGG TGCCGGACTG CCTCTCCAGC CCTGAGTTCC TCGAGCATGT
Eigenmannia_virescens            TGCGGCGTAG CGTCAGACTG CCTCTCCAGC CCTGAGTTCC TCGAGCATGT
Gymnorhamphichthys_sp.           TGCAGTGTAG TGCCCGACTG CCTCTCCAGC CCTGAGTTTC TCGAGCATGT
Microsternarchus_bilineatus      TGCGGTGTAG CGCCGGACTG TCTCTCCAGC CCTGAGTTCC TCGAACACGT
Brachyhypopomus_gauderio         TGCAGTGTAG CGCCGGACTG TCTCTCCAGC CCCGAGTTCC TCGAACATGT

                               201
Electrophorus_electricus         CACCAAGGTG ATGGTGGTGA TCGACGCAGC CGTCAACCAT CTTGATGACC
Sternopygus_macrurus             CACCAAGGTG ATGGTGGTGA TCGACGCAGC GGTCAACCAT CTTGATGACC
Apteronotus_albifrons            CACCAAGGTG ATGGTGGTGA TCGATGCAGC TGTCAACCAT CTTGATGACC
Eigenmannia_virescens            TACCAAGGTG ATGGTGGTGA TCGACGCAGC TGTCAACCAT CTTGATGACT
Gymnorhamphichthys_sp.           CACCAAGGTG ATGGTAGTGA TTGACGCAGC TGTCAACCAT CTTGATAACC
Microsternarchus_bilineatus      CACCAAGGTG ATGGTCGTGA TCGACGCAGC GGTCAACCAT CTTGATGACC
Brachyhypopomus_gauderio         CACCAAGGTG ATGGTAGTGA TCGACGCAGC TGTCAGTCAT CTTGATGACC

                               251
Electrophorus_electricus         TGCATTCTTT GGAAGACTTC TTGCTGAACC TGGGCAAGAA GCACCATGCT
Sternopygus_macrurus             TGCATTCTCT GGAAGATTTC TTGCTGAACC TGGGCAAGAA GCACCATGAT
Apteronotus_albifrons            TCCATTCTTT GGAAGACTTC TTGCTGAACC TGGGCAAGAA GCATCATGCT
Eigenmannia_virescens            TGCATTCTTT GGAAGACTTC TTGCTGAATC TGGGCAAGAA GCACCATGAT
Gymnorhamphichthys_sp.           TGCATTCTTT GGAGGACTTC TTGTTGAACT TAGGGAAGAA GCACCATGCT
Microsternarchus_bilineatus      TGCAATCTTT GGAGGACTTC TTGATGAGCC TGGGGAAGAA GCACCATGCC
Brachyhypopomus_gauderio         TGCATTCTTT GGAGGACTTC TTGATGAACC TGGGGAAGAA GCACCAGGCC

                               301
Electrophorus_electricus         GTGGGTGTTA AAACCCAGTC CTTCGCCGTG GTTGGAGAAG CCCTGCTCCA
Sternopygus_macrurus             GTTGGTGTCA AAACCCAGTC ATTCGCAGTG GTCGGAGAGG CCTTGATCCA
Apteronotus_albifrons            GTGGGTGTCA AAACCCAGTC GTTTGCTGTG GTTGGAGAGG CCCTGCTCCA
Eigenmannia_virescens            GTGGGTGTCA AGACTCAGTC CTTTGCTGTG GTGGGAGAGG CCCTGCTCCA
Gymnorhamphichthys_sp.           GTAGGTGTCA AAACCCAGTC TTTTGCTGTG GTGGGGGAGG CCCTCCTGCA
Microsternarchus_bilineatus      GTGGGTGTAA AGACCCAGTC CTTCACTGTA GTGGGTGAGG CTCTGATCCA
Brachyhypopomus_gauderio         GTGGGGGTCA AAACCCACTC CTTCACCGTG GTGGGAGAGG CCCTGCTCCA

                               351
Electrophorus_electricus         CATGCTGCAG TGCAGTCTGG GCGCCAGTTA CACCACGGCA CTGCGCCAGG
Sternopygus_macrurus             CATGCTGCAG TGCAGTCTGG GTGCCAGTTA CACGACGGCA CTGCGCCAGG
Apteronotus_albifrons            TATGCTGCAA TGCAGTCTGG GAGCAAGTTA CACGACGGCA CTGCGTCAGG
Eigenmannia_virescens            CATGCTGCAG TGCAGTCTGG GTGCGGGTTA CACCACAGCA CTGCGGCAGG
Gymnorhamphichthys_sp.           CATGCTGCAG TGCAGTCTGG GAGCGAGTTA CACTACGGCA CTGCGCCAGG
Microsternarchus_bilineatus      CATGCTGCAG TGCAGTCTGG GTGCGGGTTA CACCACGGCG CTGCGCCAGG
Brachyhypopomus_gauderio         CATGCTGCAG TGCAGTCTGG GTGCCAGTTA CACCACGGCT CTGCGCCAGG
```

```
                             401
Electrophorus_electricus     CGTGGCTCAA CATGTACAGC GTGGTGGTGT CAGCCATGAC CAGAGGCTGG
Sternopygus_macrurus         CATGGCTCAA CATGTACAGC ATAGTGGTGT CGGCCATGAC CAGAGGCTAT
Apteronotus_albifrons        CATGGCTCAA CATGTACAGC ATAGTGGTGT CAGCCATGAC CAGAGGTTGG
Eigenmannia_virescens        CATGGCTCAA CATGTACAGC ATAGTGGTGT CAGCCATGAC CAGAGGTTGG
Gymnorhamphichthys_sp.       CCTGGCTCAA CATGTACGGC ATCGTGGTGT CAGCCATGAG CCGTGGCTGG
Microsternarchus_bilineatus  CATGGCTCAA CATGTACAGC ATAGTGGTGT CGGCCATGAC CCGAGGC---
Brachyhypopomus_gauderio     CATGGCTCAA CATGTACGGC GTAGTGGTGT CGGCCATGAC CCGCGGCTGG

                             451
Electrophorus_electricus     GCCAAGAACG GAGAGCACAA ATCCAAC
Sternopygus_macrurus         GCCAAGAACG GGGAGCACAA GTCCAAC
Apteronotus_albifrons        GCCAAGAATG GGGAGCACAA GTCCAAT
Eigenmannia_virescens        GCCAAGAACG G--------- -------
Gymnorhamphichthys_sp.       GCCAAGAACG G--------- -------
Microsternarchus_bilineatus  ---------- ---------- -------
Brachyhypopomus_gauderio     GCCGTGAACG G--------- -------
```
