# Supplementary material for: Molecular evolution of globin genes in Gymnotiform electric fishes: relation to hypoxia tolerance
Source: BMC Evol Biol. 2017 Feb 13;17:51. doi: 10.1186/s12862-017-0893-3 (PMC5307702; doi:10.1186/s12862-017-0893-3)
Supplement: Additional file 2: Figure S1. — Sequences alignment of globin genes. Figure S2 The phylogenetic trees constructed from nucleotide sequences used maximum likelihood tests. (ZIP 729 kb) [file 12862_2017_893_MOESM2_ESM.zip › Fig. S2.pdf]

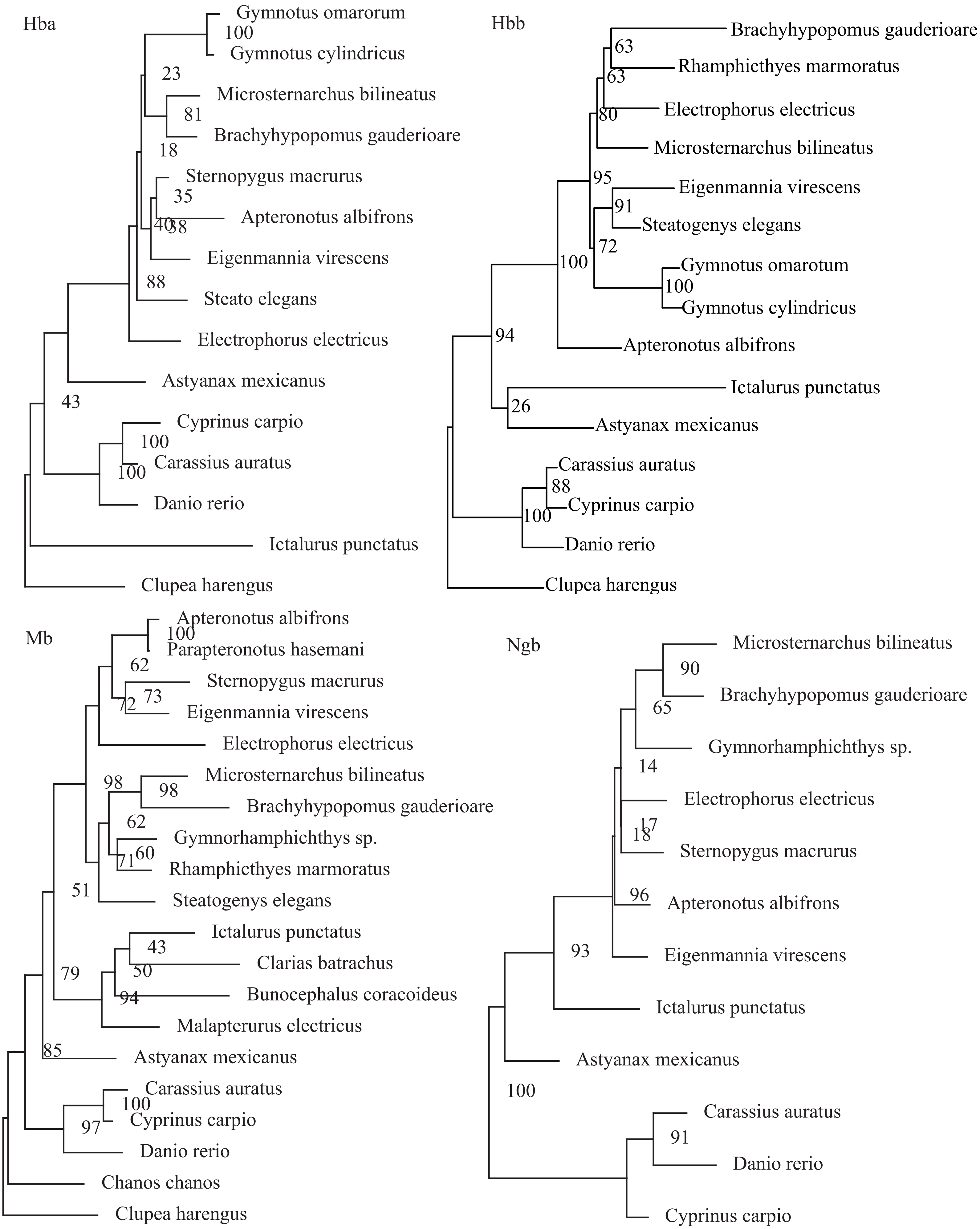

Fig. S2 The phylogenetic trees constructed from nucleotide sequences used maximum likelihood tests. Numbers on the branches indicate the bootstrap percentage values calculated from 100 replicates.
