# Supplementary material for: Molecular evolution of globin genes in Gymnotiform electric fishes: relation to hypoxia tolerance
Source: BMC Evol Biol. 2017 Feb 13;17:51. doi: 10.1186/s12862-017-0893-3 (PMC5307702; doi:10.1186/s12862-017-0893-3)
Supplement: Additional file 3: — Further details of Brachyhypopomus gauderio transcriptome. (DOCX 133 kb) [file 12862_2017_893_MOESM3_ESM.docx]

**Further details of *Brachyhypopomus gauderio* transcriptome:**

We removed tissues from a single *Brachyhypopomus gauderio* and extracted total RNA from brain, skeletal muscle, heart and electric organ. RNA was treated with RiboZero (Illumina, MRZH-11124) kit to remove ribosomal RNA, and cDNA libraries (151 bp, paired ends) were made. Libraries were sequenced on an Illumina HiSeq machine.

We processed the raw reads with Trimmomatic v0.32 (Bolger et al. 2014) for adapter removal (IlluminaClip: TruSeq3-PE.fa:2:30:10), quality trimming (SlidingWindow:4:5, Leading:5, Trailing:5) and size filtering (MinLen:25) (Table S6). These are Trinity’s default settings, which are based on the work of MacManes (2014). We performed quality control of both raw and processed reads with FastQC v0.11.3 (Babraham Bioinformatics, http://www.bioinformatics.babraham.ac.uk/).

We combined the processed PE reads across organs*, in silico* normalized, and *de novo* assembled them with Trinity v2.2.0 (Grabherr et al. 2011; Haas et al. 2013). We set min_contig_length 152 (read length + 1), with the intention of saving potential ncRNAs in the assembly. Then we used BUSCO v1.1b1 (Simão et al. 2015), along with BLAST+ v2.2.31 (Camacho et al. 2009), HMMER v3.1b1 (Finn et al. 2011), and EMBOSS v6.5.7 (Rice et al. 2000); to measure transcriptome completeness, against the Vertebrates, Metazoans and Eukaryotes datasets. In all cases, the assembly displayed a large percentage of complete orthologs (Table S7).

We estimated levels of expression for each transcript and gene, per organ, using the Trinity-provided scripts align_and_estimate_abundance.pl and abundance_estimates_to_matrix.pl. We used both RSEM v1.2.19 (Li and Dewey 2011) and kallisto v0.42.5 (Bray et al. 2016) quantification methods, which produced qualitatively very similar results. Reported abundances are TMM-normalized values calculated with the RSEM method. Only one Trinity gene blasted against the myoglobin gene sequence with an E-value of 0.00. This gene’s per organ abundances are the ones reported.

The abundance estimation results suggested that very few genes accounted for a large fraction of gene expression. Upon inspection of said genes, many were related with rRNA, and therefore were expected to be depleted during the library preparation process. Although our *Brachyhypopomus gauderio* transcriptome assembly meets our quality standards, we don’t recommend future use of the RiboZero kit (which is designed for human, mouse and rat) when working with RNAseq from this taxon.

**References:**

Bolger AM, Lohse M, Usadel B. 2014. Trimmomatic: a flexible trimmer for Illumina sequence data. *Bioinformatics*. *30*:2114–2120.

Bray NL et al. 2016. Near-optimal probabilistic RNA-seq quantification. *Nat Biotechnol.* 34:525–527.

Camacho C et al. 2009. BLAST+: architecture and applications. *BMC Bioinformatics*. 10:421.

Finn RD, Clements J, Eddy SR. 2011. HMMER web server: interactive sequence similarity searching. *Nucleic Acids Res.* 39:W29–37.

Grabherr MG et al. 2011. Full-length transcriptome assembly from RNA-Seq data without a reference genome. *Nat Biotechnol*. 29:644–52.

Haas BJ et al. 2013. De novo transcript sequence reconstruction from RNA-seq using the Trinity platform for reference generation and analysis. *Nat Protoc*. 8:1494–512.

Li B, Dewey CN. 2011. RSEM: accurate transcript quantification from RNA-Seq data with or without a reference genome. *BMC Bioinformatics*. 12:323.

MacManes MD. 2014. On the optimal trimming of high-throughput mRNA sequence data. *Frontiers Genet*. 5:1–7.

Rice P, Longden I, Bleasby A. 2000. EMBOSS: the European Molecular Biology Open Software Suite. *Trends Genet*. 16:276–7.

Simão FA et al. 2015. BUSCO: Assessing genome assembly and annotation completeness with single-copy orthologs. *Bioinformatics*. 31:3210–3212.
